# Synthesis and Evaluation of 1,8‐Disubstituted‐Cyclam/Naphthalimide Conjugates as Probes for Metal Ions

**DOI:** 10.1002/open.201600010

**Published:** 2016-08-03

**Authors:** Joseph K.‐H. Wong, Sandra Ast, Mingfeng Yu, Roman Flehr, Andrew J. Counsell, Peter Turner, Patrick Crisologo, Matthew H. Todd, Peter J. Rutledge

**Affiliations:** ^1^ School of ChemistryThe University of SydneySydneyNSW2006Australia; ^2^ Institute for ChemistryUniversity of PotsdamKarl-Liebknecht St. 24—2514476PotsdamGermany; ^3^ Crystal Structure Analysis FacilityThe University of SydneySydneyNSW2006Australia

**Keywords:** chromophores, click triazoles, fluorescent probes, macrocyclic compounds, photophysics

## Abstract

Fluorescent molecular probes for metal ions have a raft of potential applications in chemistry and biomedicine. We report the synthesis and photophysical characterisation of 1,8‐disubstituted‐cyclam/naphthalimide conjugates and their zinc complexes. An efficient synthesis of 1,8‐bis‐(2‐azidoethyl)cyclam has been developed and used to prepare 1,8‐disubstituted triazolyl‐cyclam systems, in which the pendant group is connected to triazole C4. UV/Vis and fluorescence emission spectra, zinc binding experiments, fluorescence quantum yield and lifetime measurements and pH titrations of the resultant bis‐naphthalimide ligand elucidate a complex pattern of photophysical behaviour. Important differences arise from the inclusion of two fluorophores in the one probe and from the variation of triazole substitution pattern (dye at C4 vs. N1). Introducing a second fluorophore greatly extends fluorescence lifetimes, whereas the altered substitution pattern at the cyclam amines exerts a major influence on fluorescence output and metal binding. Crystal structures of two key zinc complexes evidence variations in triazole coordination that mirror the solution‐phase behaviour of these systems.

## 1 Introduction

**Figure 1:**
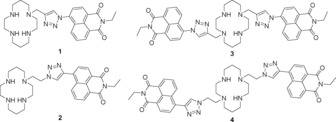
Cyclam–naphthalimide conjugates studied as fluorescent probes: the original mono‐naphthalimide compound **1**, in which the dye is connected to triazole N1;^15^ the ‘reversed’ probe **2** with the dye connected to triazole C4;^16^ bis‐naphthalimide **3** with original connectivity;^17^ and the new bis‐naphthalimide **4**, which has the reversed connectivity in this work.

The development of fluorescent molecular probes for biologically significant metal ions has attracted considerable attention in recent years.^1^, ^2^, ^3^, ^4^, ^5^, ^6^, ^7^, ^8^ Many such probes incorporate a naphthalimide fluorophore (1 *H*‐benzo[*de*]isoquinoline‐1,3(2 *H*)‐dione, Figure 1), owing to the favourable properties of this system, which include high photostability, bright fluorescence and long Stokes shifts.^9^, ^10^, ^11^, ^12^, ^13^, ^14^

We have previously reported a series of macrocyclic molecular probes based on a cyclam–triazole–naphthalimide receptor–linker–fluorophore system, with mono‐naphthalimides **1** 15 and **2** 16 and the symmetrical bis‐naphthalimide **3**.^17^ In these and related systems,^18^, ^19^, ^20^, ^21^, ^22^, ^23^, ^24^, ^25^ the triazole linker was generated by a Cu^I^‐catalysed azide–alkyne cycloaddition reaction.^26^, ^27^ The versatility of this click reaction allows the quick modular attachment of a variety of fluorophores to the tetradentate cyclam core, while also installing the coordinating triazole as a potential fifth metal‐binding site.^5^

In the original cyclam–triazole–naphthalimide probe **1**, the fluorescent dye is connected to triazole N1: ligand **1** was made by clicking a naphthalimide‐azide with a propargyl cyclam.^15^ This system demonstrated high selectivity for zinc(II) over other metal cations and a sixfold enhancement of fluorescence intensity in response to zinc binding in aqueous solution (1.0 mm HEPES buffer at pH 7). It was rationalised that, in the free ligand, photoinduced electron transfer (PET) from the cyclam/triazole unit to the naphthalimide fluorophore quenches fluorescence; when zinc binds, this PET is retarded and the fluorescence output is enhanced. Ligand **1** also showed moderate fluorescence quenching in response to copper(II) and mercury(II).^15^, ^22^

The related probe **2** was made by combining an ethynyl‐naphthalimide with an azidoethyl‐cyclam, so the dye is connected to triazole C4 in the resulting ligand.^16^ This simple reversal of triazole connectivity wrought a tenfold brighter signal in response to zinc(II), as it enables an alternative mode of fluorescence quenching; twisted intramolecular charge transfer (TICT) quenching predominates in the excited state of **2**, and is interrupted by zinc coordination.^16^, ^22^, ^28^ The emission wavelength and Stokes shift of ligand **2** vary significantly with solvent polarity: *λ*_em_=458 nm in aqueous solution versus 437 nm in acetonitrile.^16^, ^22^, ^28^ A solvent‐dependent shift in emission wavelength like this—specifically a redshift in a solvent of higher polarity—is consistent with an increase in dipole moment in the excited state and a TICT quenching mechanism with ligand **2**.^29^, ^30^

Incorporating a second triazolyl‐naphthalimide unit by combining the naphthalimide‐azide with a symmetrical bis‐propargyl cyclam afforded ligand **3**, which displayed metal‐ion responses similar to **1**: significant fluorescence enhancement in the presence of zinc(II) and moderate quenching with copper(II) or mercury(II) bound. A 12.7‐fold increase in the emission maximum of **3** was observed upon adding one equivalent of zinc(II)—double the enhancement reported with probe **1**. However, for reasons of solubility, ligand **3** was evaluated only in a mixed solvent system of water and acetonitrile (7:3, buffered at pH 7 with 50 mm HEPES buffer).^17^

We have recently observed significant solvent dependence in the fluorescence properties of ligand **2**,^16^ as noted above, and wished to revisit the fluorescence response of ligand **3** in light of this observed solvatochromaticity. Moreover, what happens when the structural properties of **2** and **3** are combined and two C4‐linked naphthalimides are incorporated in a 1,8‐disubstituted cyclam‐triazole‐naphthalimide? Herein, we report the synthesis and characterisation of the ‘reversed’ bis‐naphthalimide probe **4**, plus a more detailed photophysical investigation of bis‐naphthalimide **3**, to determine the intrinsic fluorescence properties of these probes and the basis of their response to metal‐ion binding.

## 2 Results and Discussion

### 2.1 Synthesis of 1,8‐Disubstituted‐Cyclam Derivatives

**Scheme 1:**
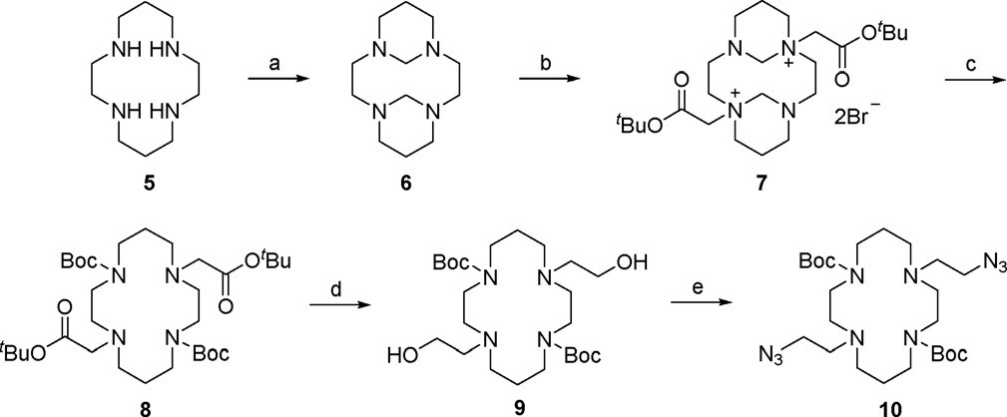
Synthesis of bis‐azido cyclam intermediate **10**: a) formaldehyde, H_2_O, 0 °C, 3 h, 89 %; b) *tert*‐butyl bromoacetate, MeCN, rt, 16 h, 81 %; c) 2.5 m NaOH, MeOH, rt, 1 h (i), Boc_2_O, 2.5 m NaOH, MeOH, rt, 16 h (ii), 87 % over steps (i) and (ii); d) LiAlH_4_, THF, 0 °C, 1 h, 99 %; e) DPPA, NaN_3_, DBU, THF, reflux, 16 h, 75 %.

**Scheme 2:**
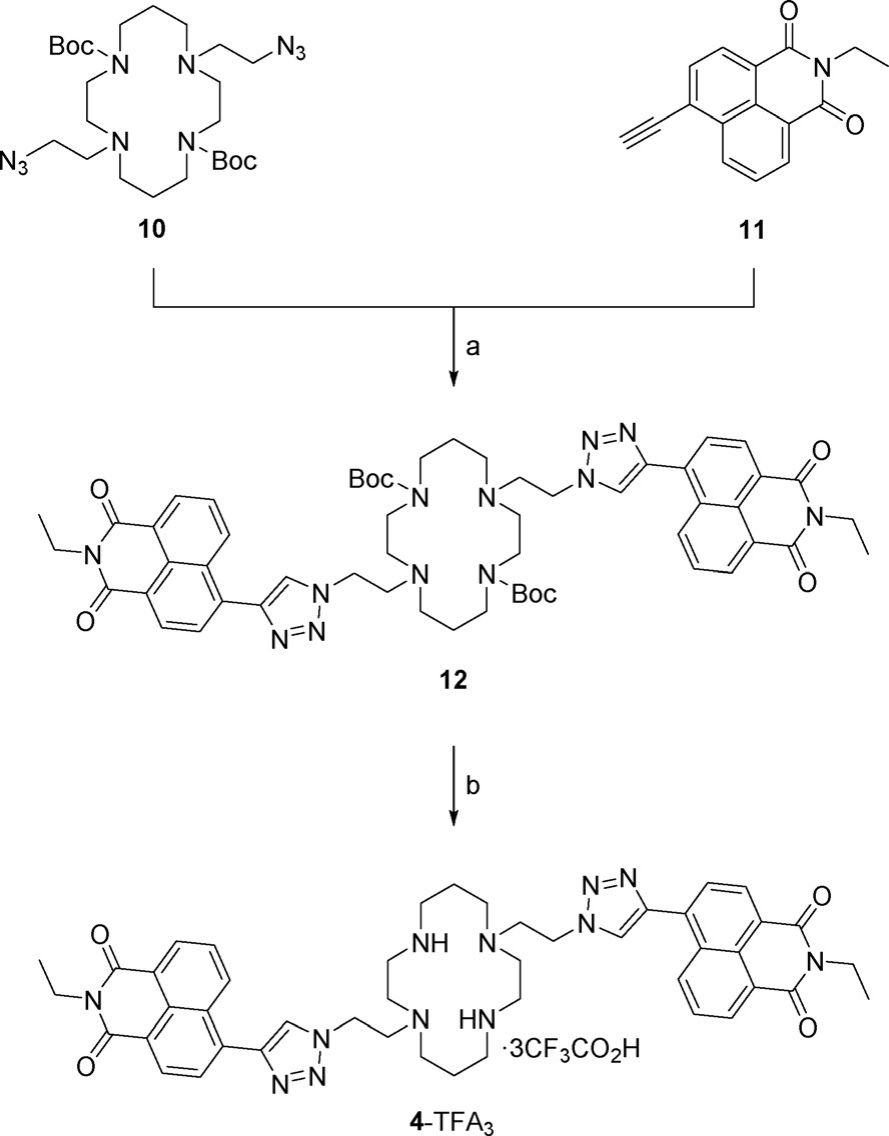
Synthesis of reversed bis‐naphthalimide probe **4**: a) CuSO_4_⋅5 H_2_O, sodium ascorbate, THF/H_2_O (7:3), 50 °C, 16 h, 93 %; b) TFA/CH_2_Cl_2_/H_2_O (90:5:5), rt, 1 h, 81 %.

A reliable route to compound **3** has been developed previously and was used to prepare ligand **3** for this work;^16^, ^17^ compound **4** has not been reported previously, and an effective synthetic path to this probe was developed as part of the current study (Schemes 1 and 2).

Cyclam **5** was first combined with formaldehyde to generate bridged derivative **6** by following a literature method.^31^ Attempts to alkylate **6** directly with toluenesulfonic acid‐2‐ azidoethyl ester (TsOCH_2_CH_2_N_3_), built on the established route to **2**,^16^, ^18^ were unsuccessful; unreacted starting material **6** was recovered. So, **6** was instead alkylated with *tert*‐butyl bromoacetate, adapting Pandya's procedure,^31^ to give the known 1,8‐*trans tert*‐butyl ester salt **7**. A high‐yielding, one‐pot procedure was developed to induce cleavage of the bis‐aminal bridges of **7** and Boc protection of the liberated secondary amines, using aqueous sodium hydroxide and di‐*tert*‐butyl dicarbonate to generate intermediate **8**. The *tert*‐butyl esters were reduced by using LiAlH_4_, giving diol **9** in high purity and near‐quantitative yield (99 %).

It had been envisaged that conversion of diol **9** to a bis‐mesylate followed by azide displacement would give the desired bis‐azide intermediate **10**. However, this sequence of reactions proved highly unreliable with yields ranging from 6 to 60 % and averaging about 30 % over the two steps. Several by‐products were observed by using TLC during the mesylation reaction, with some evidence that the reaction conditions were promoting chlorination of **9** (OHs replaced by Cls) and partial Boc deprotection. Treating diol **9** with diphenylphosphoryl azide (DPPA) and sodium azide in refluxing THF^32^ generated the desired bis‐azide **10** in high yield (75 %). Attempts to use DPPA alone—without sodium azide—were not successful and led to recovery of starting material **9**, whereas experiments using the corresponding diphenylphosphoryl chloride with sodium azide proceeded, but only with poor yield (ca. 25 %).

To complete the synthesis of **4** (Scheme 2), bis‐azide **10** was reacted with *N*‐ethyl‐4‐ethynyl‐1,8‐naphthalimide **11** 33 under modified click conditions used previously to make ligands **1**, **2** and **3**;^15^, ^16^, ^17^ this afforded Boc‐protected bis‐naphthalimide **12** in excellent yield (93 %). The Boc groups were removed by using a TFA/CH_2_Cl_2_/H_2_O (90:5:5) solvent system^34^ to give the TFA salt of ligand **4** in high yield (81 %). The salt was converted to the free amine **4**, immediately prior to spectroscopic experiments, using Ambersep 900 hydroxide form resin and a procedure that has been reported previously.^21^

**Scheme 3:**
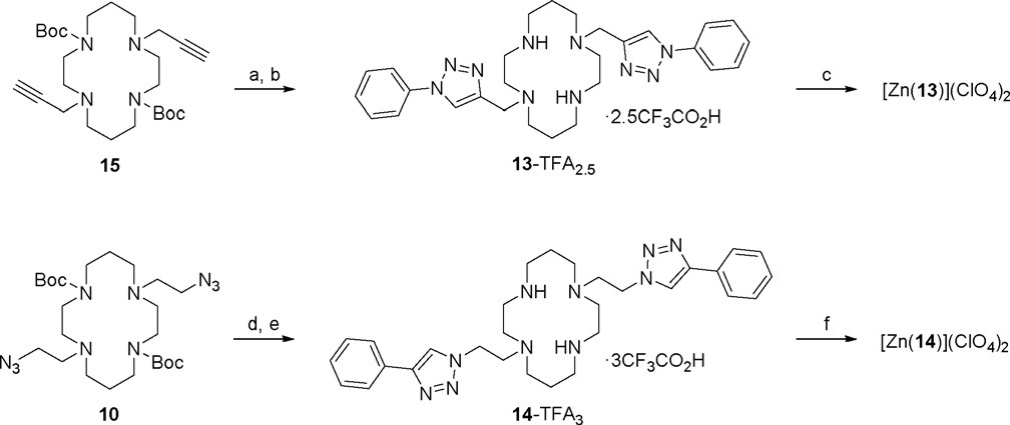
Synthesis of model compounds **13** and **14**: a) PhN_3_, CuSO_4_⋅5 H_2_O, sodium ascorbate, THF/H_2_O (7:3), 50 °C, 16 h, 61 %; b) TFA/CH_2_Cl_2_/H_2_O (90:5:5), rt, 16 h, 98 %; c) Ambersep 900 hydroxide form resin, EtOH, rt, 10 min (i), Zn(ClO_4_)_2_⋅6 H_2_O, EtOH, reflux, 16 h (ii), 41 % over steps (i) and (ii); d) PhC≡CH, CuSO_4_⋅5 H_2_O, sodium ascorbate, THF/H_2_O (7:3), 50 °C, 16 h, 74 %; e) HCl, dioxane, rt, 16 h (i), Ambersep 900 hydroxide form resin, MeOH, rt, 10 min (ii), HPLC (0.1 % TFA in H_2_O and MeCN) (iii), 69 % over steps (i)–(iii); f) Ambersep 900 hydroxide form resin, EtOH, rt, 10 min (i), Zn(ClO_4_)_2_⋅6 H_2_O, EtOH, reflux, 16 h (ii), 68 % over steps (i) and (ii).

Phenyl–triazolyl–cyclam derivatives **13** and **14** were prepared as simplified analogues of **3** and **4**, respectively, for crystallographic analysis (Scheme 3). Compound **13** was made by uniting the doubly protected, doubly propargylated cyclam **15** 17, 35 and phenylazide to afford Boc‐protected ligand **16**, followed by TFA‐mediated deprotection; compound **14** was synthesised from the cyclam‐derived bis‐azide **10** and phenylacetylene to afford Boc‐protected ligand **17**, followed by HCl‐mediated deprotection and HPLC purification. Ligands **13** and **14** were each converted to the corresponding zinc(II) complexes by adapting the method reported previously for related systems.^34^

### 2.2 Metal Binding Experiments in Aqueous Solution

**Figure 2:**
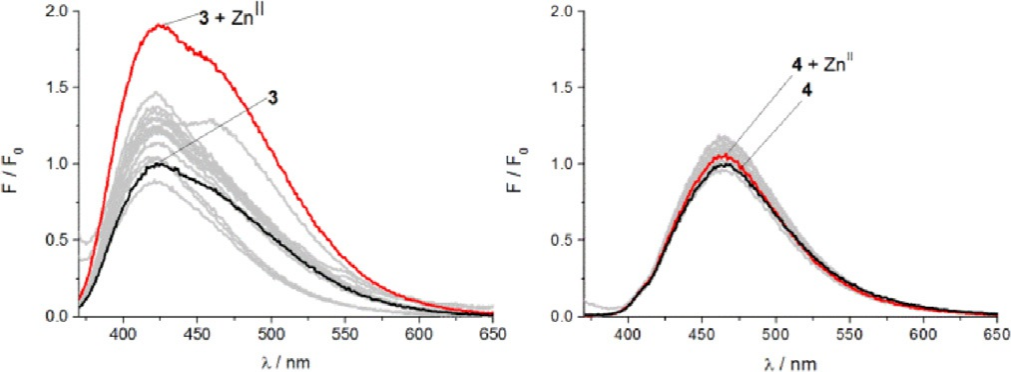
Fluorescence enhancement factors of **A**) probe **3** (2 μm, *λ*_ex_=350 nm) with different metal ions (20 μm) in HEPES buffer (10 mm, pH 7.4); **B**) probe **4** (2 μm, *λ*_ex_=357 nm) with different metal ions (20 μm) in HEPES buffer (10 mm, pH 7.4).

The response of probes **3** and **4** to a wide variety of metal ions was first evaluated in aqueous solution (10 mm HEPES buffer at pH 7.4); the ions Ag^I^, Ba^II^, Ca^II^, Cd^II^, Co^II^, Cu^II^, Fe^II^, Fe^III^, Hg^II^, K^I^, Li^I^, Mg^II^, Mn^II^, Na^I^, Ni^II^, Pb^II^, Rb^I^ and Zn^II^ were all screened with each ligand (Figure 2). Both ligands proved largely unresponsive under these conditions; both **3** and **4** gave only a weak fluorescence enhancement in the presence of Zn^II^ (red line, Figure 2) and no discernible response to either Cu^II^ or Hg^II^. This is an intriguing contrast to the mono‐naphthalimide ligands **1** and **2**, both of which exhibit a strong fluorescence enhancement in the presence of Zn^II^ (ca. six fold) and significant fluorescence quenching in the presence of Cu^II^ and Hg^II^.^15^, ^16^, ^22^

### 2.3 Metal Binding Experiments in Acetonitrile

The fluorescence response of ligands **3** and **4** to metal binding changes markedly in acetonitrile. In this solvent system, both ligands exhibit a significant turn‐on response to Zn^II^, and more detailed mechanistic studies were undertaken in this medium.

**Figure 3:**
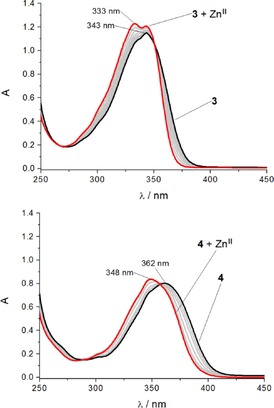
Changes in UV/Vis absorption spectra upon addition of Zn^II^: **A**) probe **3** and **B**) probe **4** (50 μm ligand solution in MeCN, with addition of Zn(ClO_4_)_2_ solution (5 mm in MeCN), added in steps of 0.1 equivalents up to total addition of 1.1 equivalents).

The UV/Vis absorption spectrum of probe **3** in acetonitrile shows the expected naphthalimide absorption maximum centred at 343 nm and a shoulder at 333 nm (black line, Figure 3 A). For reversed probe **4**, the absorption maximum is centred at 362 nm with a weak shoulder at 348 nm (black line, Figure 3 B). This redshift of 19 nm relative to **3** can be attributed to the extended aromatic system that results from the reversed triazole connectivity, in which the naphthalimide is connected to triazole C4 (versus N1 in probe **3**).^22^ Monitoring changes in the absorption spectra of ligand **3** in response to increasing Zn^II^ concentrations reveals an isosbestic point at 350 nm and significant changes in the naphthalimide band shape, as well as a slight increase in total absorbance. As the concentration of Zn^II^ approaches one equivalent, the shoulder at 333 nm transforms into a distinct peak as the new local maximum, giving rise to a final spectrum that contains two peaks at 333 and 343 nm. In contrast, the addition of Zn^II^ to probe **4** triggers a discernible blueshift of the absorption maximum from 362 to 348 nm (Figure 3 B), accompanied by an increase in naphthalimide absorbance. These changes in absorption profile give rise to an isosbestic point at 359 nm. The shape of these spectra also changes throughout the titration, indicating a conformational change of the chromophore when the ligand complexes Zn^II^.

These differences in the absorption spectra of the zinc(II) complexes of probes **3** and **4** indicate differences in the coordination behaviour of the two ligands. This could be rationalised by invoking coordination of both triazoles to the bound metal ion in the case of ligand **4**, but only ‘one‐armed’ coordination with ligand **3**, with the second pendant chromophore hanging free. To test this hypothesis, further studies were undertaken to investigate the excited state of these ligands, and to characterise the zinc complexes crystallographically.

### 2.4 Fluorescence Emission Spectra

**Figure 4:**
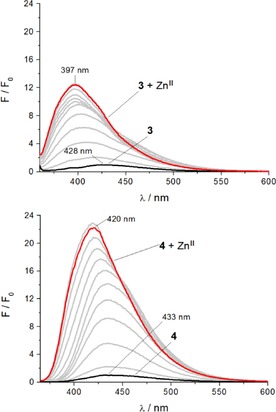
Changes in fluorescence spectra in response to increasing zinc(II) concentrations: **A**) probe **3** (*λ*_ex_=350 nm) and **B**) probe **4** (*λ*_ex_=359 nm). (2 μm ligand solution in MeCN with addition of Zn(ClO_4_)_2_ (400 μm solution in MeCN) in steps of 0.1 equivalent up to total addition of 1.1 equivalents).

Both probes **3** and **4** were titrated with increasing concentrations of Zn^II^ in acetonitrile while exciting at their respective isosbestic points (350 and 359 nm) and monitoring the fluorescence emission (Figure 4). For ligand **3**, the addition of one equivalent of Zn^II^ brought a 12.5‐fold fluorescence enhancement with a blueshift of the emission maximum, from 428 nm for **3** to 397 nm for **3**⋅Zn^II^; these spectra show an isosbestic point at 448 nm and a weak shoulder at 428 nm (Figure 4 A). With ligand **4**, on the other hand, adding one equivalent of Zn^II^ produced a 22‐fold fluorescence enhancement accompanied by a blueshift of the emission maximum, from 433 nm for **4** to 420 nm for **4**⋅Zn^II^; these spectra displayed an isosbestic point at 446 nm, formed as a result of changes to the band shape (Figure 4 B).

Plotting the integrated emission spectra versus equivalents of Zn^II^ added confirms a 1:1 stoichiometry for the complexation of **4** with Zn^II^ in acetonitrile (Figure S1). Interestingly, the corresponding plot of integrated fluorescence intensities of **3** versus Zn^II^ concentration does not show a similarly clear trend in complex formation (Figure S2). However, the 1:1 stoichiometry of the **3⋅**Zn^II^ complex was confirmed by plotting the absorption intensity at the 343 nm maximum (Figure S3). This discrepancy in outcome between the two spectroscopic methods indicates the existence of multiple excited‐state species in ligand **3**. Revisiting the shape of the fluorescence emission spectrum of **3⋅**Zn^II^ reveals that the shoulder at 428 nm could, in fact, result from non‐coordinated chromophore overlapping partially with the zinc(II) complex formed upon coordination of the other pendent arm. As a consequence, the final spectrum of the zinc(II) titration of probe **3** (Figure 4 A) represents a mixture of both free and coordinated chromophore and, therefore, fails to achieve maximal fluorescence enhancement.

The inconsistent changes in fluorescence intensity of **3** throughout the zinc(II) titration can also be rationalised by considering the presence of multiple transitions in the excited state. The changes observed in the emission profile and the blueshift of the peak maxima underline the presence of at least two chromophore‐based transitions in excited ligand **3**. The presence of two transitions of very similar energy has been shown previously by theoretical calculations of a triazolyl‐naphthalimide chromophore with N1‐connectivity.^36^ That this is seen only for ligand **3**—and not with **4**—presumably results from the different naphthalimide/triazole connectivity, as well as the strong electron‐withdrawing character of the naphthalimide itself. Thus, generating an electron‐rich centre at the naphthalimide core through the formation of an *N*‐naphthalimide bond leads to strong internal charge transfer (ICT).

### 2.5 Fluorescence Quantum Yields and Lifetimes

Fluorescence quantum yields (*Φ*) and lifetimes (*τ*) were determined for ligands **3** and **4**, and their zinc complexes **3**⋅Zn^II^ and **4**⋅Zn^II^ (Table 1), to better understand the differences in the spectroscopic features of these ligands and their different responses to zinc(II) complexation.

**Table 1:** Overview of spectral properties of probes **1**, **2**, **3** and **4**.

	*λ*_abs_ [nm]	*λ*_em_ [nm]	*Φ*	*τ* ^[b]^ [ns]
**HEPES buffer**				
**1**	347	416	0.003	0.16
**1**⋅Zn^II^	347	407	0.065	0.33
**2**	358	458	0.140	1.00
**2**⋅Zn^II^	357	435	0.760	2.02
**3**	344	424	0.066	3.36 (420 nm), 6.53 (460 nm)
**3**⋅Zn^II^	343	424, 467^[a]^	0.068	3.51 (420 nm), 6.64 (460 nm)
**4**	358	477	0.027	3.90
**4**⋅Zn^II^	358	472	0.033	3.84
				
**MeCN**				
**1**	342	406	0.003	0.22
**1**⋅Zn^II^	342	398	0.030	0.60
**2**	357	437	0.009	0.82
**2**⋅Zn^II^	357	437	0.550	3.30
**3**	333^[a]^, 343	428	0.026	3.63 (400 nm), 6.29 (420 nm)
**3**⋅Zn^II^	333, 343^[a]^	397, 428^[a]^	0.087	3.36 (400 nm), 5.89 (420 nm)
**4**	362	433	0.015	1.52 (420 nm), 2.19 (440 nm)
**4**⋅Zn^II^	348	420	0.473	3.61 (420 nm), 3.88 (440 nm)

[a] Shoulder. [b] Averaged fluorescence *τ* from the bi‐exponential decay profile.

In aqueous buffer, the quantum yield of the free ligand **3** (0.066) is more than twice that of ligand **4** (0.027). More strikingly, the *Φ* of ligand **3** is 22 times higher than that of the corresponding mono‐naphthalimide ligand **1** (0.003); not only is the *Φ* of uncomplexed **3** considerably higher than that of uncomplexed **1**, it also matches the value measured for the **1**⋅Zn^II^ complex (0.065), indicating the absence of an efficient fluorescence‐quenching process in free ligand **3**. In contrast, the quantum yield of ligand **4** (0.027) is significantly smaller than that of the corresponding mono‐naphthalimide **2** (0.140), and much below that of the **2**⋅Zn^II^ complex (0.760).

The quantum yields of the zinc(II) complexes in aqueous solution reflect the observations made in steady‐state emission studies above; zinc binding to ligands **3** and **4** triggers much smaller changes in fluorescence emission (0.066→0.068 and 0.027→0.033, respectively) compared to the corresponding mono‐naphthalimide ligands **1** and **2** (0.003→0.065 and 0.140→0.760, respectively). As discussed above, the difference in quantum yield between mono‐substituted ligand **1** and **1**⋅Zn^II^ results from suppression of PET upon zinc binding, whereas the variation between **2** and **2**⋅Zn^II^ arises primarily through a TICT mechanism.^22^ For bis‐naphthalimide probes **3** and **4**, however, the situation is not so clear. It appears that there are different effects happening in parallel, which also interfere with each other.

In acetonitrile, the quantum yield of free ligand **3** (0.026) is again higher than that of **4** (0.015), but by a lesser margin, and only around nine times higher than that of ligand **1** (0.003), reflecting qualitatively the results found in water. However, in organic solvent, the quantum yield of **4** (0.015) is higher than that of the mono‐naphthalimide analogue **2** (0.009); a direct contrast to the aqueous conditions. Zinc binding to the bis‐naphthalimide ligands triggers much more significant fluorescence enhancements in acetonitrile: *Φ* for **3**⋅Zn^II^ (0.087) is 3.3 times higher than that of uncomplexed **3** (0.026), whereas *Φ* for **4**⋅Zn^II^ (0.473) is more than 30 times higher than that of free ligand **4** (0.015). The zinc complexes of the mono‐substituted ligands **1** and **2** in acetonitrile also have higher *Φ* values than the free ligands: *Φ* for **2**⋅Zn^II^ (0.550) is more than 60 times higher than that of free ligand **2** (0.009), whereas the *Φ* increase of **1⋅**Zn^II^ (0.030) relative to free ligand **1** (0.003) is exactly tenfold. As seen in water, the quantum yield of ligand **3** (0.026) in acetonitrile is close to that of **1**⋅Zn^II^ (0.030), indicating a much weaker fluorescence quenching mechanism in free ligand **3** compared to free ligand **1**, even in the non‐protic solvent. Thus, it is apparent that appending a second fluorophore affects the efficiency of fluorescence quenching in ligand **3**, but not in ligand **4**, in which the naphthalimide is attached to triazole C4, and there is a two‐carbon spacer between cyclam and the triazole.

To investigate the nature of the excited state species in free ligand **3** and its zinc(II) complex, the fluorescence lifetime *τ* was monitored at two different wavelengths. The aim of this experiment was to determine if there are two very close transitions in the excited chromophore of this system, if—in the complex **3**⋅Zn^II^—only one of the pendant arms coordinates to the metal, or if a combination of both effects is at play. For ligand **3** in acetonitrile, this meant exciting at its emission maximum (ca. 400 nm) and at the shoulder (ca. 420 nm), which gave rise to two different fluorescence lifetimes; *τ* is 3.63 ns when exciting at 400 nm, but almost double that (6.29 ns) when exciting at 420 nm. This is strong evidence for the presence of two transitions in the excited fluorophore of **3**. The fluorescence lifetimes of the complex **3**⋅Zn^II^ are very similar to those of the free ligand: 3.36 ns at 400 nm and 5.89 ns at 420 nm. This is in contrast to the quantum yield and steady‐state emission measurements, where significant changes are apparent in response to zinc binding. This apparent contradiction supports the proposal that one fluorophore remains unaffected by zinc coordination—that is not coordinated to the metal—and suggests that this uncoordinated triazolyl‐naphthalimide unit is masking the *τ* of the complexed fluorophore in **3**⋅Zn^II^. The corresponding experiment in aqueous buffer reveals a similar result, that is, different lifetimes are observed at different excitation wavelengths, and the *τ* values recorded in aqueous solution similar to those measured in acetonitrile: 3.36 and 6.53 ns for the free ligand, 3.51 and 6.64 ns for the zinc(II) complex.

For comparison, the fluorescence lifetimes of ligand **4** were also monitored at two different wavelengths in acetonitrile, and two similar values were observed: *τ* is 1.52 ns exciting at 420 nm and 2.19 ns at 440 nm. The lifetimes of the complex **4**⋅Zn^II^ are roughly double those of the free ligand: 3.61 ns at 420 nm and 3.88 ns at 440 nm. The changes upon metal binding are qualitatively in agreement with the differences in steady‐state emission and quantum yield measurements for **4** versus **4**⋅Zn^II^, but in contrast to **3** versus **3**⋅Zn^II^.

Interestingly, attaching the second triazolyl‐naphthalimide unit to cyclam leads to longer fluorescence lifetimes for bis‐substituted probes **3** and **4** compared to the mono‐substituted analogues **1** and **2**. Appending the second chromophore brings an order of magnitude increase in *τ* in the case of **3** compared to **1** (a 21‐fold increase in aqueous solvent, 16.5‐fold in acetonitrile) and roughly doubled *τ* for **4** relative to ligand **2** (3.9 times larger in aqueous solvent, 1.9 times in acetonitrile). The lifetimes of the zinc(II) complexes are also longer for the bis‐substituted probes **3** and **4**, though less significantly so.

Thus, with ligand **3**, multiple effects are operating in the free ligand and its zinc(II) complex. The intrinsically high quantum yield of the free ligand and the fact that only one of the two triazoles coordinates to the metal upon zinc binding (vide infra) both contribute to the absence of a significant response to zinc(II) in aqueous buffer, and the relatively poor turn‐on response in acetonitrile. The presence of two very close transitions in the excited state is a possible explanation for the much higher quantum yield of **3** compared to both the mono‐substituted analogue **1** and the structurally related bis‐naphthalimide derivative **4** in both solvents. With ligand **4**, however, the quantum yield is not intrinsically high, nor is mono‐triazole coordination evident. So, the question remains: why is a more significant fluorescence turn‐on response to zinc binding not seen for ligand **4** in water?

### 2.6 pH Dependence of Zinc Response

**Figure 5:**
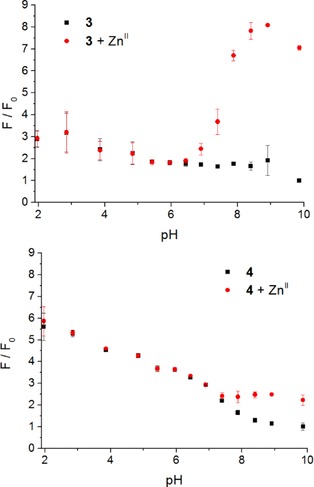
**A**) Variation of fluorescence enhancement for **3** (black squares) and **3**⋅Zn^II^ (red circles) with pH; **B**) fluorescence enhancement factors for **4** (black squares) and **4**⋅Zn^II^ (red circles) at different pHs. Error bars represent the standard deviation over **3** replicates.

To address the question raised above, pH‐dependent measurements were performed. Emission spectra of **3** and **4** in water were recorded at a range of pH values, before and after the addition of Zn^II^; the spectra were integrated and plotted against pH (Figure 5). Interestingly, both ligands as well as their Zn^II^ complexes exhibited contrasting behaviour.

The fluorescence intensity of ligand **3** is greater at lower pH values, when the cyclam nitrogen atoms are protonated; the fluorescence enhancement at pH 2 is about threefold compared to that at pH 10. When zinc(II) is added to the mix, the fluorescence output of **3**⋅Zn^II^ is greater than **3** alone at pH>6.5. At pH 9 (pH of maximal fluorescence), **3**⋅Zn^II^ exhibits an almost fourfold fluorescence enhancement over the free ligand **3**. Below pH 6.5, the addition of zinc(II) has no significant effect on the fluorescence output of **3**, suggesting that zinc complexation is inhibited in this pH range.

Ligand **4** also displays higher fluorescence enhancement at lower pH values. The fluorescence enhancement is greater with **4** than that with **3** at the corresponding acidic pH values, and this enhancement decreases more substantially at higher pH values. For ligand **4**, the fluorescence enhancement at pH 10 is about 5.5‐fold weaker than that at pH 2. Again, the addition of zinc(II) has little or no effect on the fluorescence output of **4** at acidic pH values, up to pH 7.4. Above 7.4, formation of the **4**⋅Zn^II^ complex *does* trigger an increased fluorescence output, to a maximal twofold enhancement over the free ligand at pH 9.

The variation of fluorescence intensity with pH seen with ligands **3** and **4** is consistent with previous observations when using ligand **1**,^15^ the coumarin analogue of **2**,^18^ and the anthracenyl‐cyclam fluoro‐ionophores studied by Fabbrizzi et al.^37^ Fabbrizzi et al. characterised fluorescence changes versus pH in cyclam ligands bearing an appended anthracene fluorophore, and mapped the influence of pH on metal binding. These collected observations are consistent with the postulation that PET from the amines in an uncomplexed, non‐protonated cyclam unit quenches fluorescence of the appended fluorophore, whereas protonation or metal coordination inhibits this PET and—at least partially—restores fluorescence.

Crucially, the pH at which ligand **4** begins to show a discernible response to metal‐ion binding (ca. 8.0) is a full pH unit above the equivalent value for ligand **3** (ca. 7.0), and both of these values are significantly higher than those measured previously for the corresponding mono‐naphthalimide ligand **1** (4.5),^15^ and coumarin analogue of ligand **2** (4.5), which contains the same ion‐binding motif as **2**.^18^ Thus, it appears that the low fluorescence quantum yield of ligand **4** relative to **2** in HEPES buffer at pH 7.4, and the weak response of **4** to zinc(II) binding under these conditions, are caused by the higher p*K*_a_ of the cyclam‐amines in the bis‐naphthalimide system compared to those in the mono‐naphthalimide.

### 2.7 Crystal Structure Determination

**Figure 6:**
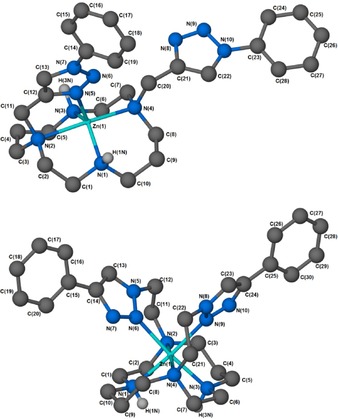
Ball‐and‐stick depictions of the crystal structures of **A**) [Zn(**13**)](ClO_4_)_2_ and **B**) [Zn(**14**)](ClO_4_)_2_ (exogenous perchlorates omitted for clarity) generated with X‐Seed^38^ and POV‐Ray.^39^ Non‐hydrogen‐bonding hydrogen atoms omitted.

The significant change in absorption behaviour of **3** in the presence of increasing concentrations of zinc(II) highlights the possibility of additional conformational changes in the ground state. Attempts to study these conformational changes using ^1^H NMR spectroscopy were hampered by the different solubility profiles of the free ligands **3** and **4** compared to the complexes **3**⋅Zn^II^ and **4**⋅Zn^II^, and the fact that the NMR spectra of the zinc complexes are complicated by the aza‐macrocycle adopting multiple geometries upon metal binding (see Sections 14 and 15 in the Supporting Information). Thus, single‐crystal X‐ray diffraction studies of the analogous 1,8‐disubstituted cyclam–triazole–phenyl model systems **13** and **14** were undertaken. Ball‐and‐stick depictions of the resultant structures [Zn(**13**)](ClO_4_)_2_ and [Zn(**14**)](ClO_4_)_2_ are shown in Figure 6; crystallographic data (Table S1), selected bond lengths (Table S2) and ORTEP depictions (Figure S4 and S5) are presented in the Supporting Information.^40^

The [Zn(**13**)](ClO_4_)_2_ complex (Figure 6 A) adopts a strained trigonal bipyramidal pentacoordinate geometry around the zinc(II) centre, typical of previously reported zinc(II) cyclams.^17^, ^41^ The orientation of the macrocyclic *N*‐substituents indicates that complex [Zn(**13**)](ClO_4_)_2_ adopts the *trans*‐I configuration;^42^ this being the preferred stereochemistry of pentacoordinate cyclam complexes.^43^, ^44^ Importantly, this structure reveals that *only one* of the triazole‐phenyl arms of the bis‐substituted system coordinates to the metal centre. It does so with N3 of the pendant 1,2,3‐triazole [N(5) in the crystallographic numbering scheme] coordinating to the metal ion at an equatorial position. The equatorial coordination bond lengths are 2.049(3) Å [Zn(1)−N(1)], 2.056(3) Å [Zn(1)−N(3)], and 2.043(3) Å [Zn(1)−N(5)] and are close to the calculated ideal of 2.07 Å.^45^

The axial positions of [Zn(**13**)](ClO_4_)_2_ are occupied by macrocyclic nitrogens N(2) and N(4), with an N(2)−Zn(1)−N(4) angle of 174.06(12)° and bond lengths of 2.287(3) Å [Zn(1)−N(2)] and 2.196(3) Å [Zn(1)−N(4)]. These metrics are consistent with previous observations that M−N distances are generally longer for tertiary than for secondary amines in 3d‐metal complexes.^46^ The observed Zn–triazole interaction is consistent with the prominent shift in the absorption maximum observed as the Zn titration of **3** proceeds (Figure 3 A), owing to a change in the composition of the combined transitions at 333 and 343 nm, which arise from the presence of both mono‐coordinated and free naphthalimide side‐chain species.

Compound [Zn(**14**)](ClO_4_)_2_ contains the reversed triazole connectivity. This complex exhibits the more unusual *cis*‐V configuration of the macrocycle, with the zinc(II) adopting a distorted octahedral coordination geometry. The structure displays mutual *cis* coordination of *both* pendant triazole‐phenyl arms, consistent with the photophysical observations made using the corresponding naphthalimide ligand **4** (Figure 3 B). The reversed connectivity imposes a difference in the nature of triazole coordination; in contrast to the triazole N3 coordination observed in [Zn(**13**)](ClO_4_)_2_ and reflecting greater ligand flexibility, in [Zn(**14**)](ClO_4_)_2_, the triazole coordinates through N2. We have previously found the same behaviour in copper(II) and mercury(II) complexes of related compounds.^18^

We have also previously reported a crystal structure for the bis‐triazolyl‐benzyl pendant analogue of [Zn(**13**)](ClO_4_)_2_—where benzyl replaces phenyl—in which the macrocycle adopts a *trans*‐III configuration and *both* triazole pendants are coordinated to the metal through triazole N3.^17^ Moreover, the two triazoles occupy the opposite axial sites within an octahedral coordination sphere. Comparison with the structures of [Zn(**13**)](ClO_4_)_2_ and [Zn(**14**)](ClO_4_)_2_ reported here suggests that the electronics of the triazole are important in determining the mode of coordination, as much as the nature of the linker between the macrocycle and the triazole (i.e. whether *n*=1 or 2 in the cyclam–(CH_2_)_*n*_–triazole connection).

Further discussion of these crystal structures and their relationship to others previously described elsewhere is presented in the Supporting Information (Section 7).

## 3 Conclusions

An efficient route to 1,8‐bis‐(2‐azidoethyl)cyclam **10** has been developed and used to prepare 1,8‐disubstituted cyclam systems containing the ‘reversed’ triazole topology, that is, dye/pendant group connected to triazole C4 (versus N1 in the ‘original’ systems). Photophysical characterisation of bis‐naphthalimide ligands **3** and **4** and their responses to metal ions reveal complex behaviour and important differences between the two, and between these ligands and the corresponding mono‐naphthalimides **1** and **2**. Notably, introducing the second chromophore greatly extends fluorescence lifetimes of **3** relative to **1**, and **4** relative to **2**. Also, differences in the basicity of the triazole and cyclam nitrogen atoms with changing substitution exert a significant influence on fluorescence output and metal binding. Single‐crystal X‐ray structures of zinc complexes of bis‐phenyl‐triazolyl analogues **13** and **14** show that, although both triazoles coordinate to the metal in the ‘reversed’ compound **14**, only one is bound to zinc in **13**, which are consistent with the photophysical behaviour of these systems in solution.

## Experimental Section

**Safety note**: Sodium azide, organic azides and perchlorate salts of metal complexes with organic ligands are potentially explosive. Only small amounts of material should be prepared and these should be handled with caution.

General experimental procedures are detailed in the Supporting Information.

### 1 ,4,8,11‐Tetraazatricyclo[9.3.1.1(4,8)]hexadecane, 6

Formaldehyde (37 %, 2.23 mL, 27.5 mmol) was added to a chilled solution of cyclam **5** (1.00 g, 4.99 mmol) in H_2_O (50 mL). The reaction mixture was stirred at 0 °C for 3 h. The product was filtered and washed with H_2_O (100 mL) to yield **6** as a white solid (1.00 g, 89 %). m.p.: 105–107 (lit. 106–108 °C)^47^. ^1^H NMR (500 MHz, CDCl_3_): *δ*=1.13–1.21 (m, 2 H), 2.24 (dt, 1 H, *J* 12.9, 5.2), 2.29 (dt, 1 H, *J* 13.0, 5.2), 2.38 (d, 4 H, *J* 10.0), 2.62 (td, 4 H, *J* 12.6, 3.6), 2.81–2.87 (m, 4 H), 2.90 (d, 2 H, *J* 10.9), 3.14 (d, 4 H, *J* 9.9), 5.44 ppm (dt, 2 H, *J* 10.9, 2.1). LRMS (ESI^+^): *m/z* 225.1 ([M+H]^+^, 100 %). The spectroscopic data were in agreement with those in the literature.^35^, ^47^

### 2 ,8‐Bis(2‐(*tert*‐butoxy)‐2‐oxoethyl)‐1,4,8,11‐tetraazatricyclo[9.3.1.1(4,8)]hexadecane‐1,8‐diium Dibromide, 7

*tert*‐Butyl bromoacetate (1.87 mL, 12.7 mmol) was added to a solution of **6** (1.13 g, 5.06 mmol) in MeCN (10 mL). The reaction mixture was stirred at rt for 16 h. The product was isolated by centrifugation and washed with MeCN (20 mL) to yield **7** as a white solid (2.51 g, 81 %). m.p.: 185 °C (decomp.). ^1^H NMR (400 MHz, [D_6_]DMSO): *δ*=1.49 (s, 18 H), 1.70–1.85 (m, 2 H), 2.31–2.48 (m, 4 H), 2.72 (d, 2 H, *J* 15.2), 3.03–3.14 (m, 2 H), 3.25 (d, 2 H, *J* 13.6), 3.36 (d, 2 H, *J* 15.2), 3.50–3.61 (m, 2 H), 3.64 (d, 2 H, *J* 9.7), 3.81 (d, 2 H, *J* 11.4), 4.35 (t, 2 H, *J* 13.9), 4.43 (d, 2 H, *J* 16.7), 4.59 (d, 2 H, *J* 16.7), 5.23 ppm (d, 2 H, *J* 9.6). ^13^C NMR (100 MHz, [D_6_]DMSO): *δ*=19.2, 27.6, 46.4, 47.7, 50.6, 57.3, 59.9, 76.5, 84.3, 163.5 ppm. LRMS (ESI+): *m/z* 429.0 [M‐2 CH_2_‐2 Br+3 H]^+^. The NMR data were in agreement with those in the literature;^31^ the melting point of this compound has not been reported previously.

### Di‐*tert*‐butyl 4,11‐bis(2‐(*tert*‐butoxy)‐2‐oxoethyl)‐1,4,8,11‐ tetraazacyclotetradecane‐1,8‐dicarboxylate, 8

NaOH (2.5 m, 10.0 mL, 25.0 mmol) was added to a solution of **7** (2.40 g, 3.91 mmol) in MeOH (10 mL). The reaction mixture was stirred at rt for 1 h before the addition of a solution of di‐*tert*‐butyl dicarbonate (2.98 g, 13.7 mmol) in MeOH (5 mL) and stirring was continued at rt for 16 h. MeOH was removed, and the product was filtered and washed with H_2_O (100 mL) to afford the Boc‐protected product **8** as an off‐white solid (2.13 g, 87 %). m.p.: 103–104 °C. ^1^H NMR (400 MHz, CDCl_3_): *δ*=1.44 (s, 18 H), 1.45 (s, 18 H), 1.66–1.75 (m, 4 H), 2.65 (t, 4 H, *J* 5.5), 2.79 (t, 4 H, *J* 5.5), 3.24 (s, 4 H), 3.28 (br s, 4 H), 3.40 ppm (br s, 4 H). ^13^C NMR (100 MHz, CDCl_3_): *δ*=27.2, 28.2, 28.5, 46.4, 47.1, 52.3, 53.4, 57.3, 79.2, 80.8, 155.7, 170.7 ppm. LRMS (ESI+): *m/z* 629.5 ([M+H]^+^, 100 %). HRMS (ESI+): *m/z* Calcd. for C_32_H_61_N_4_O_8_^+^ [M+H]^+^ 629.4484, found 629.4481. FTIR (ATR) *ν*_max_ cm^−1^: 2976, 2932, 1732, 1691, 1410, 1366, 1250, 1155.

### Di‐*tert*‐butyl 4,11‐bis(2‐hydroxyethyl)‐1,4,8,11‐tetraazacyclotetradecane‐1,8‐dicarboxylate, 9

LiAlH_4_ (1.0 m, 4.40 mL, 4.40 mmol) was added to a chilled solution of **8** (694 mg, 1.10 mmol) in THF (10 mL). The reaction mixture was stirred at 0 °C for 1 h and quenched by the sequential slow addition of EtOAc (1 mL) and H_2_O (1 mL). Rochelle salt (sat., 10 mL) was added and the volatiles were removed. The product was extracted with EtOAc (2×25 mL), and the extracts were combined, dried (Na_2_SO_4_) and concentrated under reduced pressure to afford the bis‐alcohol **9** as a yellowish oil (532 mg, 99 %). The crude product was used in subsequent reactions without purification. ^1^H NMR (400 MHz, CDCl_3_): *δ*=1.46 (s, 18 H), 1.77 (qn, 4 H, *J* 6.9), 2.49 (t, 4 H, *J* 6.2), 2.57 (t, 4 H, *J* 5.2), 2.62 (t, 4 H, *J* 6.0), 3.25–3.40 (m, 8 H), 3.57 ppm (t, 4 H, *J* 5.2). ^13^C NMR (100 MHz, CDCl_3_): *δ*=27.5, 28.4, 47.2, 47.5, 52.1, 53.9, 56.9, 59.0, 79.6, 155.9 ppm. LRMS (ESI+): *m/z* 489.1 ([M+H]^+^, 41 %), 577.1 ([M+Na]^+^, 100 %). HRMS (ESI+): *m/z* Calcd. for C_24_H_49_N_4_O_6_^+^ [M+H]^+^ 489.3647, found 489.3650. FTIR (ATR) *ν*_max_ cm^−1^: 3418, 2972, 2934, 2813, 1672, 1479, 1415, 1366, 1249, 1158, 1046.

### Di‐*tert*‐butyl 4,11‐bis(2‐azidoethyl)‐1,4,8,11‐tetraazacyclo‐ tetradecane‐1,8‐dicarboxylate, 10

DPPA (1.33 mL, 6.19 mmol) and DBU (771 μL, 5.16 mmol) were added to a solution of **9** (1.26 g, 2.58 mmol) in THF (5 mL) under N_2_. The reaction mixture was stirred for 5 min and sodium azide (1.01 g, 15.5 mmol) was added. The reaction mixture was stirred at reflux for 16 h. H_2_O (75 mL) was added and the product was extracted with EtOAc (3×50 mL). The extracts were combined, dried (Na_2_SO_4_) and concentrated under reduced pressure, and the residue was purified by automated flash column chromatography (25 g cartridge, 10 % EtOAc in petroleum ether (PE) over 1 column volume (CV), 10 % to 100 % over 12 CV, 100 % over 1 CV) to afford the bis‐azide **10** as a pale yellow oil (1.04 g, 75 %). ^1^H NMR (500 MHz, CDCl_3_): *δ*=1.46 (s, 18 H), 1.68–1.80 (m, 4 H), 2.49 (t, 4 H, *J* 5.8), 2.57–2.65 (m, 4 H), 2.63 (t, 4 H, *J* 6.1), 3.26 (t, 4 H, *J* 5.8), 3.23–3.33 (m, 4 H), 3.33–3.45 ppm (m, 4 H). ^13^C NMR (100 MHz, CDCl_3_): *δ*=27.2, 28.5, 46.3, 47.1, 49.3, 52.8, 53.5, 55.1, 79.4, 155.7 ppm. LRMS (ESI+): *m/z* 539.4 ([M+H]^+^, 100 %). HRMS (ESI+): *m/z* Calcd. for C_24_H_47_N_10_O_4_^+^ [M+H]^+^ 539.3776, found 539.3774. FTIR (ATR) *ν*_max_ cm^−1^: 2972, 2934, 2822, 2098, 1690, 1470, 1410, 1391, 1250, 1157.

### Di‐*tert*‐butyl 4,11‐bis(2‐(4‐(2‐ethyl‐1,3‐dioxo‐2,3‐dihydro‐1 *H*‐benzo[*de*]isoquinolin‐6‐yl)‐1 *H*‐1,2,3‐triazol‐1‐yl)ethyl)‐1,4,8,11‐tetraazacyclotetradecane‐1,8‐dicarboxylate, 12

A mixture of copper sulfate pentahydrate (9.27 mg, 371 μmol) and sodium ascorbate (14.7 mg, 74.3 μmol) in H_2_O (3 mL) was added to a solution of **10** (200 mg, 371 μmol) and *N*‐ethyl‐4‐ethynyl‐1,8‐naphthalimide **11** (222 mg, 891 μmol) in THF (7 mL) under N_2_. The reaction mixture was stirred at 50 °C for 16 h. NH_4_Cl (sat., 25 mL) was added and the solvent was removed. The product was extracted with CH_2_Cl_2_ (2×50 mL), and the extracts were combined, dried (Na_2_SO_4_) and concentrated under reduced pressure. The residue was purified by flash column chromatography (EtOAc:PE=3:1 to EtOAc) to afford the clicked product **12** as an orange gum (357 mg, 93 %). ^1^H NMR (400 MHz, CDCl_3_): *δ*=1.34, (t, 6 H, *J* 7.1), 1.46 (s, 18 H), 1.58 (qn, 4 H, *J* 6.8), 2.36–2.47 (m, 4 H), 2.55–2.65 (m, 4 H), 2.90–3.01 (m, 4 H), 3.13–3.29 (m, 8 H), 4.24 (q, 4 H, *J* 7.1), 4.30–4.41 (m, 4 H), 7.68–7.75 (m, 2 H), 7.93–7.99 (m, 2 H), 7.97 (s, 2 H), 8.56 (d, 2 H, *J* 7.1), 8.61 (d, 2 H, *J* 7.6), 9.07 ppm (d, 2 H, *J* 8.5). ^13^C NMR (100 MHz, CDCl_3_): *δ*=13.3, 26.9, 28.5, 35.6, 46.6, 47.0, 48.8, 52.7, 54.0, 55.0, 79.8, 122.5, 122.8, 124.4, 127.0, 127.3, 128.8, 129.0, 130.6, 131.4, 132.7, 134.0, 145.6, 155.8, 163.7, 163.9 ppm. LRMS (ESI+): *m/z* 1037.5 ([M+H]^+^, 100 %), 1059.5 ([M+Na]^+^, 15 %). HRMS (ESI+): *m/z* Calcd. for C_56_H_68_N_12_NaO_8_^+^ [M+Na]^+^ 1059.5175, found 1059.5181. FTIR (ATR) *ν*_max_ cm^−1^: 3398, 2963, 1652, 1575, 1260, 1089, 1033.

### 3 ,6′‐(1,1′‐((1,4,8,11‐Tetraazacyclotetradecane‐1,8‐diyl)bis‐ (ethane‐2,1‐diyl))bis‐ (1 *H*‐1,2,3‐triazole‐4,1‐diyl))bis(2‐ ethyl‐1 *H*‐benzo[de]isoquinoline‐1,3(2 H)‐dione), 4

The Boc‐protected bis‐naphthalimide **12** (52 mg, 50 μmol) was dissolved in a mixture of TFA (4.5 mL), H_2_O (0.25 mL) and CH_2_Cl_2_ (0.25 mL), and stirred at rt for 1 h. The volatiles were removed in vacuo, and the residue was triturated with EtOAc (2×3 mL) and lyophilised to afford the TFA salt of **4** as a yellow solid (48 mg, 81 %). m.p.: 126 °C (decomp.). ^1^H NMR (500 MHz, CD_3_OD): *δ*=1.29 (t, 6 H, *J* 7.1), 2.05–2.13 (m, 4 H), 2.88 (t, 4 H, *J* 5.2), 3.00 (t, 4 H, *J* 4.8), 3.26 (t, 4 H, *J* 5.9), 3.32–3.37 (m, 4 H), 3.36 (t, 4 H, *J* 5.1), 4.14 (q, 4 H, *J* 7.1), 4.72 (t, 4 H, *J* 5.9), 7.56 (dd, 2 H, *J* 8.5, 7.3), 7.78 (d, 2 H, *J* 7.7), 8.24 (d, 2 H, *J* 7.6), 8.30 (d, 2 H, *J* 7.1), 8.47 (s, 2 H), 8.62 ppm (d, 2 H, *J* 8.4). ^13^C NMR (125 MHz, CD_3_OD): *δ*=13.5, 24.5, 36.4, 45.7, 47.6, 50.3, 52.5, 52.9, 117.8 (q, *J*_C−F_ 289.5), 123.0, 123.5, 127.1, 128.0, 128.3, 129.2, 129.5, 131.2, 131.8, 132.9, 134.7, 146.4, 162.4 ppm (q, *J*_C−F_ 36.0), 164.5, 164.8. LRMS (ESI+): *m/z* 837.3 (free base [M+H]^+^, 100 %). HRMS (ESI+): *m/z* Calcd. for C_46_H_53_N_12_O_4_^+^ free base [M+H]^+^ 837.4307, found 837.4297. FTIR (ATR) *ν*_max_ cm^−1^: 1695, 1656, 1589, 1454, 1373, 1202, 1139, 1066. Anal.: Calcd. for C_46_H_52_N_12_O_4_⋅3 CF_3_CO_2_H⋅H_2_O: C 52.17, H 4.80, N 14.04; found C 52.32, H 4.77, N 14.03.

### Di‐*tert*‐butyl 4,11‐bis((1‐phenyl‐1 *H*‐1,2,3‐triazol‐4‐yl)methyl)‐1,4,8,11‐tetraazacyclotetradecane‐1,8‐dicarboxylate, 16

A mixture of copper sulfate pentahydrate (13.0 mg, 0.0521 mmol) and sodium ascorbate (20.6 mg, 0.104 mmol) in H_2_O (3 mL) was added to a solution of bis‐propargyl cyclam **15** (280 mg, 0.520 mmol) and phenyl azide (127 mg, 1.24 mmol) in THF (7 mL) under N_2_. The reaction mixture was stirred at 50 °C for 16 h. NH_4_Cl (sat., 25 mL) was added and the solvent was removed. The product was extracted with CH_2_Cl_2_ (2×50 mL), and extracts were combined, dried (Na_2_SO_4_) and concentrated under reduced pressure. The residue was purified by automated flash chromatography (10 g cartridge, 25 % EtOAc in PE over 2 CV, 25 % to 100 % over 4 CV, 100 % over 8 CV) to afford the clicked product **16** as an off‐white solid (236 mg, 61 %). m.p.: 168–170 °C. ^1^H NMR (400 MHz, [D_6_]DMSO, 323 K) *δ*=1.30 (s, 18 H), 1.66–1.80 (m, 4 H), 2.44–2.52 (m, 4 H), 2.53–2.62 (m, 4 H), 3.27–3.39 (m, 8 H), 3.78 (s, 4 H), 7.46 (t, 2 H, *J* 7.4), 7.56 (t, 4 H, *J* 7.8), 7.85 (d, 4 H, *J* 7.7), 8.53 ppm (s, 2 H). ^13^C NMR (100 MHz, [D_6_]DMSO, 323 K) *δ*=26.4, 27.8, 45.7, 46.0, 48.7, 51.2, 52.6, 78.0, 119.7, 121.4, 128.2, 129.6, 136.6, 144.5, 154.7 ppm. LRMS (ESI+): *m/z* 715.2 ([M+H]^+^, 100 %). HRMS (ESI+): *m/z* Calcd. for C_38_H_55_N_10_O_4_^+^ [M+H]^+^ 715.4402, found 715.4400. FTIR (ATR) *ν*_max_ cm^−1^: 2971, 2930, 2812, 1687, 1503, 1468, 1414, 1366, 1233, 1156, 1042, 759. *NMR spectra were acquired at 323 K due to broadening of signals at room temperature (300 K)*.

### 4 ,8‐Bis((1‐phenyl‐1 *H*‐1,2,3‐triazol‐4‐yl)methyl)‐1,4,8,11‐ tetraazacyclotetradecane, 13

The Boc‐protected bis‐phenyl **16** (68 mg, 95 μmol) was dissolved in a mixture of TFA (4.5 mL), H_2_O (0.25 mL) and CH_2_Cl_2_ (0.25 mL), and stirred at rt for 16 h. The volatiles were removed in vacuo and lyophilisation afforded the TFA salt of **13** as an off‐white hygroscopic solid (75 mg, 98 %). ^1^H NMR (400 MHz, [D_6_]DMSO, 323 K): *δ*=2.30–2.40 (m, 4 H), 3.19 (t, 4 H, *J* 5.5), 3.31 (t, 4 H, *J* 5.5), 3.60 (br s, 4 H), 3.71 (br s, 4 H), 4.24 (s, 4 H), 7.80–8.00 (m, 10 H), 8.51 ppm (s, 2 H). ^13^C NMR (100 MHz, [D_6_]DMSO, 323 K): *δ*=23.0, 45.6, 47.6, 49.1, 51.9, 55.0, 121.4, 123.0, 130.2, 130.6, 136.7, 145.1 ppm. LRMS (ESI+): *m/z* 515.5 (free base [M+H]^+^, 100 %). HRMS (ESI+): *m/z* Calcd. for C_28_H_39_N_10_^+^ free base [M+H]^+^ 515.3354, found 515.3357. FTIR (ATR) *ν*_max_ cm^−1^: 2841, 1675, 1503, 1466, 1195, 1133, 1053, 799, 761, 721. Anal.: Calcd. for C_28_H_38_N_10_⋅2.5 CF_3_CO_2_H⋅0.5 H_2_O: C 49.01, H 5.17, N 17.32; found C 49.19, H 5.05, N 17.27. *NMR spectra were acquired at 323 K due to broadening of signals at room temperature (300 K)*.

### [Zn(13)](ClO_4_)_2_

The TFA salt of **13** (20 mg, 26 μmol) was stirred in a suspension of Ambersep 900 hydroxide form resin in EtOH (4 mL) for 10 min. The resin was filtered off and Zn(ClO_4_)_2_⋅6 H_2_O (27 mg, 26 μmol) was added. The reaction mixture was stirred at reflux for 16 h. The product was isolated by centrifugation, washed with EtOH (2×5 mL) and dried in vacuo. The product was then re‐dissolved in MeCN (5 mL) and the solution filtered through a 0.2 μm PTFE syringe filter. The solvent was removed and the product was lyophilised to afford the zinc complex of **13** as a white solid (8.5 mg, 41 %). m.p.: 282 °C (decomp.). LRMS (ESI+): *m/z* 576.9 ([M+H]^+^, 100 %). HRMS (ESI+): Calcd. for C_28_H_37_N_10_Zn^+^ [M‐2 ClO_4_‐H]^+^ 577.2490, found 577.2489. FTIR (ATR) *ν*_max_ cm^−1^: 2925, 2878, 1597, 1502, 1461, 1093, 764, 691, 623. Anal.: Calcd. for C_28_H_38_Cl_2_N_10_O_8_Zn⋅0.5 H_2_O C 42.68, H 4.99, N 17.78; found C 42.88, H 4.94, N 17.48.

### Di‐*tert*‐butyl 4,11‐bis(2‐(4‐phenyl‐1 *H*‐1,2,3‐triazol‐1‐yl)ethyl)‐1,4,8,11‐tetraazacyclotetradecane‐1,8‐dicarboxylate, 17

A mixture of copper sulfate pentahydrate (11.1 mg, 0.0445 mmol) and sodium ascorbate (17.7 mg, 0.0893 mmol) in H_2_O (3 mL) was added to a solution of **10** (240 mg, 0.446 mmol) and phenyl acetylene (117 μL, 1.07 mmol) in THF (7 mL). The reaction mixture was stirred at 50 °C for 16 h. NH_4_Cl (sat., 25 mL) was added and the solvent was removed. The product was extracted with EtOAc (2×25 mL), and the extracts were combined, dried (Na_2_SO_4_) and concentrated under reduced pressure. The residue was purified by automated flash chromatography (10 g cartridge, 10 % EtOAc in PE over 1 CV, 10 % to 100 % over 6 CV, 100 % over 5 CV) to afford the clicked product **17** as a yellowish gum (246 mg, 74 %). ^1^H NMR (400 MHz, CDCl_3_): *δ*=1.35–1.50 (m, 4 H), 1.46 (s, 18 H), 2.31 (t, 4 H, *J* 6.6), 2.47–2.59 (m, 4 H), 2.80 (t, 4 H, *J* 5.9), 3.00–3.22 (m, 8 H), 4.10–4.25 (m, 4 H), 7.27–7.34 (m, 2 H), 7.37–7.44 (m, 4 H), 7.73 (s, 2 H), 7.88 ppm (d, 4 H, *J* 7.5). ^13^C NMR (100 MHz, CDCl_3_): *δ*=26.7, 28.5, 46.4, 47.2, 48.5, 52.6, 54.2, 55.3, 79.7, 121.2, 125.6, 128.1, 128.9, 130.7, 147.1, 155.9 ppm. LRMS (ESI+): *m/z* 743.0 ([M+H]^+^, 35 %), 765.2 ([M+Na]^+^, 100 %). HRMS (ESI+): *m/z* Calcd. for C_40_H_59_N_10_O_4_^+^ [M+H]^+^ 743.4715, found 743.4716. FTIR (ATR) *ν*_max_ cm^−1^: 2973, 2817, 1684, 1466, 1414, 1365, 1248, 1230, 1161, 766, 696.

### 5 ,8‐Bis(2‐(4‐phenyl‐1 *H*‐1,2,3‐triazol‐1‐yl)ethyl)‐1,4,8,11‐ tetraazacyclotetradecane, 14

The protected bis‐phenyl **17** (243 mg, 0.327 mmol) was dissolved in dioxane (2 mL) and a solution of HCl in dioxane (4.0 m, 1.00 mL, 4.00 mmol) was added. The reaction mixture was stirred at rt for 16 h. The volatiles were removed in vacuo and the residue was triturated with EtOAc (2×3 mL). The HCl salt was neutralised with excess Ambersep 900 hydroxide form resin in MeOH (3 mL) and purified by HPLC (0 % to 100 % MeCN in H_2_O containing 0.1 % TFA over 30 min) to afford the TFA salt of **14** as a white solid (173 mg, 69 %). m.p.: 213 °C (decomp.). ^1^H NMR (400 MHz, D_2_O): *δ*=1.65–1.80 (m, 4 H), 2.35–2.49 (m, 8 H), 2.49–2.60 (m, 4 H), 2.90–3.05 (m, 8 H), 4.57 (t, 4 H, *J* 5.7), 7.36–7.44 (m, 2 H), 7.44–7.52 (m, 4 H), 7.76–7.82 (m, 4 H), 8.39 ppm (s, 2 H). ^13^C NMR (100 MHz, D_2_O): *δ*=23.1, 42.8, 43.0, 46.0, 47.0, 49.0, 49.7, 123.2, 125.2, 128.7, 129.2, 129.4, 147.6 ppm. LRMS (ESI+): *m/z* 543.1 (free base [M+H]^+^, 100 %). HRMS (ESI+) *m/z* Calcd. for C_30_H_43_N_10_^+^ free base [M+H]^+^ 543.3667, found 543.3665. FTIR (ATR) *ν*_max_ cm^−1^: 2856, 1682, 1424, 1199, 1172, 1119, 1073, 829, 770, 718, 695. Anal.: Calcd. for C_30_H_42_N_10_⋅3 CF_3_CO_2_H⋅3 H_2_O: C 48.70, H 5.45, N 15.78; found C 48.50, H 5.35, N 16.06.

### [Zn(14)](ClO_4_)_2_

The TFA salt of **14** (94 mg, 0.11 mmol) was stirred in a suspension of Ambersep 900 hydroxide form resin in EtOH (4 mL) for 10 min. The resin was filtered and Zn(ClO_4_)_2_⋅6 H_2_O (40 mg, 0.11 mg) was added. The reaction mixture was stirred at reflux for 16 h. The product was isolated by centrifugation, washed with EtOH (2×5 mL) and dried in vacuo. The product was then re‐dissolved in MeCN (5 mL) and the solution filtered through a 0.2 μm PTFE syringe filter. The solvent was removed and the product was lyophilised to afford the zinc complex of **14** as a white solid (58 mg, 68 %). m.p.: 214 °C (decomp.). LRMS (ESI+): *m/z* 705.2 ([M+H]+, 100 %). HRMS (ESI+): Calcd. for C_30_H_42_ClN_10_O_4_Zn^+^ [M‐ClO_4_]^+^ 705.2365, found 705.2354. FTIR (ATR) *ν*_max_ cm^−1^: 3260, 2875, 1453, 1358, 1331, 1067, 976, 930, 838, 768, 695, 621. Anal.: Calcd. for C_30_H_42_Cl_2_N_10_O_8_Zn C 44.65, H 5.25, N 17.36; found C 44.57, H 5.19, N 17.27.

## Supporting information

As a service to our authors and readers, this journal provides supporting information supplied by the authors. Such materials are peer reviewed and may be re‐organized for online delivery, but are not copy‐edited or typeset. Technical support issues arising from supporting information (other than missing files) should be addressed to the authors.

Supplementary
