# Supplementary material for: Synthesis and Evaluation of 1,8‐Disubstituted‐Cyclam/Naphthalimide Conjugates as Probes for Metal Ions
Source: ChemistryOpen. 2016 Aug 3;5(4):375–85. doi: 10.1002/open.201600010 (PMC4981059; doi:10.1002/open.201600010)
Supplement: Supplementary file 1 — Supplementary [file OPEN-5-375-s001.pdf]

ChemistryOPEN

Supporting Information

© 2016 The Authors. Published by Wiley-VCH Verlag GmbH & Co. KGaA, Weinheim

Synthesis and Evaluation of 1,8-Disubstituted-Cyclam/ Naphthalimide Conjugates as Probes for Metal Ions

Joseph K.-H. Wong,[a] Sandra Ast,[a] Mingfeng Yu,[a] Roman Flehr,[b] Andrew J. Counsell,[a] Peter Turner,[a, c] Patrick Crisologo,[a] Matthew H. Todd,*[a] and Peter J. Rutledge*[a]

open_201600010_sm_miscellaneous_information.pdf

Contents

1. General materials and instrumentation S2
2. General experimental procedures for photophysical investigations S3
3. Synthetic experimental procedures S3
4. Additional absorption and fluorescence data for **3** and **4** S9
5. Crystal structure determination for [Zn(**13**)](ClO_4)$_2$ and [Zn(**14**)](ClO_4)$_2$ S10
6. Crystallographic parameters for [Zn(**13**)](ClO_4)$_2$ and [Zn(**14**)](ClO_4)$_2$ S11
7. ORTEP depictions of [Zn(**13**)](ClO_4)$_2$ and [Zn(**14**)](ClO_4)$_2$ S13
8. Further Discussion of Crystal Structures S15
9. ^{1}H and ^{13}C NMR spectra of **8** S16
10. ^{1}H and ^{13}C NMR spectra of **9** S17
11. ^{1}H and ^{13}C NMR spectra of **10** S18
12. ^{1}H and ^{13}C NMR spectra of **12** S19
13. ^{1}H and ^{13}C NMR spectra of **4** S20
14. Comparison of ^{1}H NMR spectra of **3** and **3·Zn** **S**21
15. Comparison of ^{1}H NMR spectra of **4** and **4·Zn** S21
16. References S22

1. General materials and instrumentation

Acetonitrile, methanol and THF were collected fresh from a PureSolv MD 7 solvent purification system having been passed through anhydrous alumina columns. All commercially available reagents and solvents were purchased from Sigma Aldrich, Alfa Aesar, Merck or Ajax Finechem and used without purification. Flash column chromatography was performed on Davisil Grace Davison 40–63 µm (230–400 mesh) silica gel. Automated flash column chromatography was performed on a Biotage Isolera Spektra One using Biotage SNAP KP-Sil cartridges at their default flow rates. Preparative reversed-phase HPLC was carried out on a Waters 600 controller with a Waters 600 pump and a 2998 photodiode array detector. A Waters SunFire™ C18 OBD™ preparative column (5 µm, 19 × 150 mm) was used at a flow rate of 7 mL/min; mobile phases of 0.1% TFA in Milli-Q water and 0.1% TFA in acetonitrile in different ratios were used.

Melting points were recorded on a Stanford Research Systems Optimelt automated melting system and are uncorrected. ^{1}H and ^{13}C NMR spectra were recorded on a Bruker Avance DPX 400 or 500 spectrometer. Chemical shifts are reported in ppm relative to tetramethylsilane or residual solvent resonance as internal standard. Coupling constants *J* are reported in Hz to the nearest 0.1 Hz. Mass spectra (LRMS) were acquired on a Thermo Classic LCQ mass spectrometer or a Bruker amaZon SL mass spectrometer. Accurate mass measurements (HRMS) were performed on a Bruker Apex Qe 7T Fourier Transform Ion Cyclotron Resonance (FT-ICR) mass spectrometer. Infrared spectra were recorded on a Bruker ALPHA FT-IR spectrophotometer (ZnSe or diamond ATR). Elemental analyses were carried out by the Campbell Microanalytical Laboratory (University of Otago, New Zealand) on a Carlo Erba EA 1108 elemental analyser or by the Chemical Analysis Facility (Macquarie University, Australia) on a PelkinElmer PE2400 CHNS/O elemental analyser.

Fluorescence emission spectra were recorded at 25°C on a Varian Cary Eclipse spectrophotometer. UV/Vis absorption spectra were recorded at 25°C on a Varian Cary 4000 spectrophotometer. Temperature control for both spectrophotometers was provided by a Varian Cary PCB water Peltier system. For pH dependence studies, solutions of HEPES buffer (10 mM) were adjusted to the appropriate pH with $HClO_4$ or NaOH. In time-resolved experiments, samples were excited using the frequency-doubled output of Titan sapphire laser (Tsunami 3960; Spectra Physics), with the repetition rate lowered from 80.2 to 3.8 MHz using a Pulse Picker (Pulse Select; APE). Luminescence was detected at 90° to the incoming beam using a multichannel plate (ELDI EM1-132/300; Europhoton GmbH) coupled to a fluorescence lifetime spectrometer (FL920, Edinburgh Instruments). Time-resolved emissions were recorded in time-correlated single photon counting mode. Fluorescence decay was analysed using the FAST software package (Edinburgh Instruments). A PL Quantum Yield Measurement System C9920-02 with an integrating sphere (Hamamatsu) was used to record fluorescence quantum yields.

2. General experimental procedures for photophysical investigations

Metal ion screen

To a solution (2 mL) of ligand (2 µM) in *solvent* (10 mM HEPES buffer, pH 7.4 or MeCN) in a 1 cm quartz cuvette was added a solution (20 µL) of metal perchlorate hydrate (20 µM) in *solvent*. The solution was stirred for 5 minutes before fluorescence spectra were recorded.

UV/Vis titration

To a solution (2 mL) of ligand (50 µM) in MeCN in a 1 cm quartz cuvette were cumulatively added aliquots (2 µL, 0.1 equiv.) of a solution of zinc perchlorate hexahydrate (5 mM) in MeCN. The solutions were stirred for 5 minutes before absorption spectra were recorded.

Fluorescence titration

To a solution (2 mL) of ligand (2 µM) in MeCN in a 1 cm quartz cuvette were cumulatively added aliquots (0.4 µL, 0.1 equiv.) of a solution of zinc perchlorate hexahydrate (400 µM) in MeCN. The solutions were stirred for 5 minutes before fluorescence spectra were recorded. The wavelength of the observed isosbestic point from the ligands' respective UV/Vis titration spectra was chosen as the excitation wavelength. The fluorescence spectra were corrected by their dilution factors.

Fluorescence response to pH

A stock solution (1 mL) of the ligand (800 µM) in DMSO was prepared. In a 1 cm quartz cuvette, an aliquot (7.5 µL) of the stock solution was diluted to 3 mL with HEPES buffer (10 mM, different pHs adjusted from pH 7.4 solution) and to this diluted solution (2 µM) was added a solution (12 µL, 2 equiv.) of $Zn(ClO_4)_2{\cdot}6H_2O$ (1 mM) in MilliQ H_2O. Fluorescence spectra were obtained before and after the addition of Zn^{II}.

3. Synthetic experimental procedures

Safety note: *Sodium azide, organic azides and perchlorate salts of metal complexes with organic ligands are potentially explosive. Only small amounts of material should be prepared and these should be handled with caution.*

1,4,8,11-Tetraazatricyclo[9.3.1.1^{4,8}]hexadecane ***6***

To a chilled solution of cyclam **5** (1.00 g, 4.99 mmol) in H_2O (50 mL) was added formaldehyde (37%, 2.23 mL, 27.5 mmol). The reaction mixture was stirred at 0 °C for 3 h. The product was filtered and washed with H_2O (100 mL) to yield **6** as a white solid (1.00 g, 89%). **m.p.**: 105 – 107 (lit.[1] 106 – 108 °C). **^{1}H NMR**

(500 MHz, $CDCl_3$): *δ* 1.13 – 1.21 (m, 2H), 2.24 (dt, 1H, *J* 12.9, 5.2), 2.29 (dt, 1H, *J* 13.0, 5.2), 2.38 (d, 4H, *J* 10.0), 2.62 (td, 4H, *J* 12.6, 3.6), 2.81 – 2.87 (m, 4H), 2.90 (d, 2H, *J* 10.9), 3.14 (d, 4H, *J* 9.9), 5.44 (dt, 2H, *J* 10.9, 2.1). **LRMS** (ESI+): *m/z* 225.1 ($[M+H]^+$, 100%). The spectroscopic data were in agreement with those in the literature.[1-2]

1,8-Bis(2-(tert-butoxy)-2-oxoethyl)-1,4,8,11-tetraazatricyclo[9.3.1.1^{4,8}]hexadecane-1,8-diium dibromide ***7***

To a solution of **6** (1.13 g, 5.06 mmol) in MeCN (10 mL) was added *tert*-butyl bromoacetate (1.87 mL, 12.7 mmol). The reaction mixture was stirred at rt for 16 h. The product was isolated by centrifugation and washed with MeCN (20 mL) to yield **7** as a white solid (2.51 g, 81%). **m.p.**: 185 °C (decomp.). **^{1}H NMR** (400 MHz, DMSO-d_6): *δ* 1.49 (s, 18H), 1.70 – 1.85 (m, 2H), 2.31 – 2.48 (m, 4H), 2.72 (d, 2H, *J* 15.2), 3.03 – 3.14 (m, 2H), 3.25 (d, 2H, *J* 13.6), 3.36 (d, 2H, *J* 15.2), 3.50 – 3.61 (m, 2H), 3.64 (d, 2H, *J* 9.7), 3.81 (d, 2H, *J* 11.4), 4.35 (t, 2H, *J* 13.9), 4.43 (d, 2H, *J* 16.7), 4.59 (d, 2H, *J* 16.7), 5.23 (d, 2H, *J* 9.6). **^{13}C NMR** (100 MHz, DMSO-d_6): *δ* 19.2, 27.6, 46.4, 47.7, 50.6, 57.3, 59.9, 76.5, 84.3, 163.5. **LRMS** (ESI+): *m/z* 429.0 $[M\text{-}2CH_2\text{-}2Br+3H]^+$. The NMR data were in agreement with those in the literature;[3] the melting point of this compound has not been reported previously.

Di-tert-butyl 4,11-bis(2-(tert-butoxy)-2-oxoethyl)-1,4,8,11-tetraazacyclotetradecane-1,8-dicarboxylate ***8***

To a solution of **7** (2.40 g, 3.91 mmol) in MeOH (10 mL) was added NaOH (2.5 M, 10.0 mL, 25.0 mmol). The reaction mixture was stirred at rt for 1 h before the addition of a solution of di-*tert*-butyl dicarbonate (2.98 g, 13.7 mmol) in MeOH (5 mL) and stirring was continued at rt for 16 h. MeOH was removed, and the product was filtered and washed with H_2O (100 mL) to afford the Boc protected product **8** as an off-white solid (2.13 g, 87%). **m.p.**: 103 – 104 °C. **^{1}H NMR** (400 MHz, $CDCl_3$): *δ* 1.44 (s, 18H), 1.45 (s, 18H), 1.66 – 1.75 (m, 4H), 2.65 (t, 4H, *J* 5.5), 2.79 (t, 4H, *J* 5.5), 3.24 (s, 4H), 3.28 (br s, 4H), 3.40 (br s, 4H). **^{13}C NMR** (100 MHz, $CDCl_3$): *δ* 27.2, 28.2, 28.5, 46.4, 47.1, 52.3, 53.4, 57.3, 79.2, 80.8, 155.7, 170.7. **LRMS** (ESI+): *m/z* 629.5 ($[M+H]^+$, 100%). **HRMS** (ESI+): *m/z* Calcd. for $C_{32}H_{61}N_4O_8^+$ $[M+H]^+$ 629.4484, found 629.4481. **FTIR** (ATR) ν_{max}/cm^{-1}: 2976, 2932, 1732, 1691, 1410, 1366, 1250, 1155.

Di-tert-butyl 4,11-bis(2-hydroxyethyl)-1,4,8,11-tetraazacyclotetradecane-1,8-dicarboxylate ***9***

To a chilled solution of **8** (694 mg, 1.10 mmol) in THF (10 mL) was added $LiAlH_4$ (1.0 M, 4.40 mL, 4.40 mmol). The reaction mixture was stirred at 0 °C for 1 h and quenched by the sequential slow addition of EtOAc (1 mL) and H_2O (1 mL). Rochelle salt (sat., 10 mL) was added and the volatiles were removed. The product was extracted with EtOAc (2 × 25 mL), and the extracts were combined, dried (Na_2SO_4) and concentrated under reduced pressure to afford the bis-alcohol **9** as a yellowish oil (532 mg, 99%). The crude product was used in subsequent reactions without purification. **^{1}H NMR** (400 MHz, $CDCl_3$): *δ* 1.46 (s, 18H), 1.77 (qn, 4H, *J* 6.9), 2.49 (t, 4H, *J* 6.2), 2.57 (t, 4H, *J* 5.2), 2.62 (t, 4H, *J* 6.0), 3.25 – 3.40 (m, 8H), 3.57 (t, 4H, *J* 5.2). **^{13}C NMR** (100 MHz, $CDCl_3$): *δ* 27.5, 28.4, 47.2, 47.5, 52.1, 53.9, 56.9, 59.0, 79.6, 155.9. **LRMS** (ESI+): *m/z* 489.1 ($[M+H]^+$, 41%), 577.1 ($[M+Na]^+$, 100%). **HRMS** (ESI+): *m/z* Calcd. for

$C_{24}H_{49}N_4O_6^+$ [M+H]$^+$ 489.3647, found 489.3650. **FTIR** (ATR) ν_{max}/cm^{-1}: 3418, 2972, 2934, 2813, 1672, 1479, 1415, 1366, 1249, 1158, 1046.

Di-tert-butyl 4,11-bis(2-azidoethyl)-1,4,8,11-tetraazacyclotetradecane-1,8-dicarboxylate ***10***

To a solution of **9** (1.26 g, 2.58 mmol) in THF (5 mL) were added DPPA (1.33 mL, 6.19 mmol) and DBU (771 µL, 5.16 mmol) under N_2. The reaction mixture was stirred for 5 min and sodium azide (1.01 g, 15.5 mmol) was added. The reaction mixture was stirred at reflux for 16 h. H_2O (75 mL) was added and the product was extracted with EtOAc (3 × 50 mL). The extracts were combined, dried (Na_2SO_4) and concentrated under reduced pressure, and the residue was purified by automated flash column chromatography (25 g cartridge, 10% EtOAc in P. E. over 1 column volume (CV), 10% to 100% over 12 CV, 100% over 1 CV) to afford the bis-azide **10** as a pale yellow oil (1.04 g, 75%). **^{1}H NMR** (500 MHz, $CDCl_3$): δ 1.46 (s, 18H), 1.68 – 1.80 (m, 4H), 2.49 (t, 4H, *J* 5.8), 2.57 – 2.65 (m, 4H), 2.63 (t, 4H, *J* 6.1), 3.26 (t, 4H, *J* 5.8), 3.23 – 3.33 (m, 4H), 3.33 – 3.45 (m, 4H). **^{13}C NMR** (100 MHz, $CDCl_3$): δ 27.2, 28.5, 46.3, 47.1, 49.3, 52.8, 53.5, 55.1, 79.4, 155.7. **LRMS** (ESI+): *m/z* 539.4 ([M+H]$^+$, 100%). **HRMS** (ESI+): *m/z* Calcd. for $C_{24}H_{47}N_{10}O_4^+$ [M+H]$^+$ 539.3776, found 539.3774. **FTIR** (ATR) ν_{max}/cm^{-1}: 2972, 2934, 2822, 2098, 1690, 1470, 1410, 1391, 1250, 1157.

Di-tert-butyl 4,11-bis(2-(4-(2-ethyl-1,3-dioxo-2,3-dihydro-1H-benzo[de]isoquinolin-6-yl)-1H-1,2,3-triazol-1-yl)ethyl)-1,4,8,11-tetraazacyclotetradecane-1,8-dicarboxylate ***12***

To a solution of **10** (200 mg, 371 µmol) and *N*-ethyl-4-ethynyl-1,8-naphthalimide **11** (222 mg, 891 µmol) in THF (7 mL) was added a mixture of copper sulfate pentahydrate (9.27 mg, 371 µmol) and sodium ascorbate (14.7 mg, 74.3 µmol) in H_2O (3 mL) under N_2. The reaction mixture was stirred at 50 °C for 16 h. NH_4Cl (sat., 25 mL) was added and the solvent was removed. The product was extracted with CH_2Cl_2 (2 × 50 mL), and the extracts were combined, dried (Na_2SO_4) and concentrated under reduced pressure. The residue was purified by flash column chromatography (EtOAc:P. E. = 3:1 to EtOAc) to afford the clicked product **12** as an orange gum (357 mg, 93%). **^{1}H NMR** (400 MHz, $CDCl_3$): δ 1.34, (t, 6H, *J* 7.1), 1.46 (s, 18H), 1.58 (qn, 4H, *J* 6.8), 2.36 – 2.47 (m, 4H), 2.55 – 2.65 (m, 4H), 2.90 – 3.01 (m, 4H), 3.13 – 3.29 (m, 8H), 4.24 (q, 4H, *J* 7.1), 4.30 – 4.41 (m, 4H), 7.68 – 7.75 (m, 2H), 7.93 – 7.99 (m, 2H), 7.97 (s, 2H), 8.56 (d, 2H, *J* 7.1), 8.61 (d, 2H, *J* 7.6), 9.07 (d, 2H, *J* 8.5). **^{13}C NMR** (100 MHz, $CDCl_3$): δ 13.3, 26.9, 28.5, 35.6, 46.6, 47.0, 48.8, 52.7, 54.0, 55.0, 79.8, 122.5, 122.8, 124.4, 127.0, 127.3, 128.8, 129.0, 130.6, 131.4, 132.7, 134.0, 145.6, 155.8, 163.7, 163.9. **LRMS** (ESI+): *m/z* 1037.5 ([M+H]$^+$, 100%), 1059.5 ([M+Na]$^+$, 15%). **HRMS** (ESI+): *m/z* Calcd. for $C_{56}H_{68}N_{12}NaO_8^+$ [M+Na]$^+$ 1059.5175, found 1059.5181. **FTIR** (ATR) ν_{max}/cm^{-1}: 3398, 2963, 1652, 1575, 1260, 1089, 1033.

6,6'-(1,1'-((1,4,8,11-Tetraazacyclotetradecane-1,8-diyl)bis(ethane-2,1-diyl))bis(1H-1,2,3-triazole-4,1-diyl))bis(2-ethyl-1H-benzo[de]isoquinoline-1,3(2H)-dione) ***4***

The Boc-protected bis-naphthalimide **12** (52 mg, 50 µmol) was dissolved in a mixture of TFA (4.5 mL), H_2O (0.25 mL) and CH_2Cl_2 (0.25 mL), and stirred at rt for 1 h. The volatiles were removed *in vacuo,* and the residue was triturated with EtOAc (2 x 3 mL) and lyophilised to afford the TFA salt of **4** as a yellow solid (48 mg, 81%). **m.p.**: 126 °C (decomp.). **^{1}H NMR** (500 MHz, CD_3OD): δ 1.29 (t, 6H, J 7.1), 2.05 – 2.13 (m, 4H), 2.88 (t, 4H, J 5.2), 3.00 (t, 4H, J 4.8), 3.26 (t, 4H, J 5.9), 3.32 – 3.37 (m, 4H), 3.36 (t, 4H, J 5.1), 4.14 (q, 4H, J 7.1), 4.72 (t, 4H, J 5.9), 7.56 (dd, 2H, J 8.5, 7.3), 7.78 (d, 2H, J 7.7), 8.24 (d, 2H, J 7.6), 8.30 (d, 2H, J 7.1), 8.47 (s, 2H), 8.62 (d, 2H, J 8.4). **^{13}C NMR** (125 MHz, CD_3OD): δ 13.5, 24.5, 36.4, 45.7, 47.6, 50.3, 52.5, 52.9, 117.8 (q, $J_{C\text{-}F}$ 289.5), 123.0, 123.5, 127.1, 128.0, 128.3, 129.2, 129.5, 131.2, 131.8, 132.9, 134.7, 146.4, 162.4 (q, $J_{C\text{-}F}$ 36.0), 164.5, 164.8. **LRMS** (ESI+): *m/z* 837.3 (free base $[M+H]^+$, 100%). **HRMS** (ESI+): *m/z* Calcd. for $C_{46}H_{53}N_{12}O_4^+$ free base $[M+H]^+$ 837.4307, found 837.4297. **FTIR** (ATR) ν_{max}/cm^{-1}: 1695, 1656, 1589, 1454, 1373, 1202, 1139, 1066. **Anal.**: Calcd. for $C_{46}H_{52}N_{12}O_4 \cdot 3CF_3CO_2H \cdot H_2O$: C 52.17, H 4.80, N 14.04; found C 52.32, H 4.77, N 14.03.

*Di-tert-butyl 4,11-bis((1-phenyl-1H-1,2,3-triazol-4-yl)methyl)-1,4,8,11-tetraazacyclotetradecane-1,8-dicarboxylate **16***

To a solution of bis-propargyl cyclam **15** (280 mg, 0.520 mmol) and phenyl azide (127 mg, 1.24 mmol) in THF (7 mL) was added a mixture of copper sulfate pentahydrate (13.0 mg, 0.0521 mmol) and sodium ascorbate (20.6 mg, 0.104 mmol) in H_2O (3 mL) under N_2. The reaction mixture was stirred at 50 °C for 16 h. NH_4Cl (sat., 25 mL) was added and the solvent was removed. The product was extracted with CH_2Cl_2 (2 × 50 mL), and extracts were combined, dried (Na_2SO_4) and concentrated under reduced pressure. The residue was purified by automated flash chromatography (10 g cartridge, 25% EtOAc in P. E. over 2 CV, 25% to 100% over 4 CV, 100% over 8 CV) to afford the clicked product **16** as an off-white solid (236 mg, 61%). **m.p**.: 168-170 °C. **^{1}H NMR** (400 MHz, DMSO-d_6, 323 K) δ 1.30 (s, 18H), 1.66-1.80 (m, 4H), 2.44 – 2.52 (m, 4H), 2.53 – 2.62 (m, 4H), 3.27 – 3.39 (m, 8H), 3.78 (s, 4H), 7.46 (t, 2H, J 7.4), 7.56 (t, 4H, J 7.8), 7.85 (d, 4H, J 7.7), 8.53 (s, 2H). **^{13}C NMR** (100 MHz, DMSO-d_6, 323 K) δ 26.4, 27.8, 45.7, 46.0, 48.7, 51.2, 52.6, 78.0, 119.7, 121.4, 128.2, 129.6, 136.6, 144.5, 154.7. **LRMS** (ESI+): *m/z* 715.2 ($[M+H]^+$, 100%). **HRMS** (ESI+): *m/z* Calcd. for $C_{38}H_{55}N_{10}O_4^+$ $[M+H]^+$ 715.4402, found 715.4400. **FTIR** (ATR) ν_{max}/cm^{-1}: 2971, 2930, 2812, 1687, 1503, 1468, 1414, 1366, 1233, 1156, 1042, 759. *NMR spectra were acquired at 323 K due to broadening of signals at room temperature (300 K)*

*1,8-Bis((1-phenyl-1H-1,2,3-triazol-4-yl)methyl)-1,4,8,11-tetraazacyclotetradecane **13***

The Boc-protected bis-phenyl **16** (68 mg, 95 µmol) was dissolved in a mixture of TFA (4.5 mL), H_2O (0.25 mL) and CH_2Cl_2 (0.25 mL), and stirred at rt for 16 h. The volatiles were removed *in vacuo* and lyophilisation afforded the TFA salt of **13** as an off-white hygroscopic solid (75 mg, 98%). **^{1}H NMR** (400 MHz, DMSO-d_6, 323 K): δ 2.30 – 2.40 (m, 4H), 3.19 (t, 4H, J 5.5), 3.31 (t, 4H, J 5.5), 3.60 (br s, 4H), 3.71 (br s, 4H), 4.24 (s, 4H), 7.80 – 8.00 (m, 10H), 8.51 (s, 2H). **^{13}C NMR** (100 MHz, DMSO-d_6, 323 K): δ 23.0,

45.6, 47.6, 49.1, 51.9, 55.0, 121.4, 123.0, 130.2, 130.6, 136.7, 145.1. **LRMS** (ESI+): *m/z* 515.5 (free base $[M+H]^+$, 100%). **HRMS** (ESI+): *m/z* Calcd. for $C_{28}H_{39}N_{10}^+$ free base $[M+H]^+$ 515.3354, found 515.3357. **FTIR** (ATR) ν_{max}/cm^{-1}: 2841, 1675, 1503, 1466, 1195, 1133, 1053, 799, 761, 721. **Anal.**: Calcd. for $C_{28}H_{38}N_{10}\cdot 2.5CF_3CO_2H\cdot 0.5H_2O$: C 49.01, H 5.17, N 17.32; found C 49.19, H 5.05, N 17.27. *NMR spectra were acquired at 323 K due to broadening of signals at room temperature (300 K)*

*[Zn(**13**)](ClO$_4$)$_2$*

The TFA salt of **13** (20 mg, 26 µmol) was stirred in a suspension of Ambersep® 900 hydroxide form resin in EtOH (4 mL) for 10 min. The resin was filtered off and $Zn(ClO_4)_2\cdot 6H_2O$ (27 mg, 26 µmol) was added. The reaction mixture was stirred at reflux for 16 h. The product was isolated by centrifugation, washed with EtOH (2 × 5 mL) and dried *in vacuo*. The product was then redissolved in MeCN (5 mL) and the solution filtered through a 0.2 µm PTFE syringe filter. The solvent was removed and the product was lyophilised to afford the zinc complex of **13** as a white solid (8.5 mg, 41%). **m.p.**: 282 ºC (decomp.). **LRMS** (ESI+): *m/z* 576.9 ($[M+H]^+$, 100%). **HRMS** (ESI+): Calcd. for $C_{28}H_{37}N_{10}Zn^+$ $[M-2ClO_4-H]^+$ 577.2490, found 577.2489. **FTIR** (ATR) ν_{max}/cm^{-1}: 2925, 2878, 1597, 1502, 1461, 1093, 764, 691, 623. **Anal.**: Calcd. for $C_{28}H_{38}Cl_2N_{10}O_8Zn\cdot 0.5H_2O$ C 42.68, H 4.99, N 17.78; found C 42.88, H 4.94, N 17.48.

*Di-tert-butyl 4,11-bis(2-(4-phenyl-1H-1,2,3-triazol-1-yl)ethyl)-1,4,8,11-tetraazacyclotetradecane-1,8-dicarboxylate **17***

To a solution of **10** (240 mg, 0.446 mmol) and phenyl acetylene (117 µL, 1.07 mmol) in THF (7 mL) was added a mixture of copper sulfate pentahydrate (11.1 mg, 0.0445 mmol) and sodium ascorbate (17.7 mg, 0.0893 mmol) in H_2O (3 mL). The reaction mixture was stirred at 50 °C for 16 h. NH_4Cl (sat., 25 mL) was added and the solvent was removed. The product was extracted with EtOAc (2 × 25 mL), and the extracts were combined, dried (Na_2SO_4) and concentrated under reduced pressure. The residue was purified by automated flash chromatography (10 g cartridge, 10% EtOAc in P. E. over 1 CV, 10% to 100% over 6 CV, 100% over 5 CV) to afford the clicked product **17** as a yellowish gum (246 mg, 74%). **^{1}H NMR** (400 MHz, $CDCl_3$): δ 1.35 – 1.50 (m, 4H), 1.46 (s, 18H), 2.31 (t, 4H, *J* 6.6), 2.47 – 2.59 (m, 4H), 2.80 (t, 4H, *J* 5.9), 3.00 – 3.22 (m, 8H), 4.10 – 4.25 (m, 4H), 7.27 – 7.34 (m, 2H), 7.37 – 7.44 (m, 4H), 7.73 (s, 2H), 7.88 (d, 4H, *J* 7.5). **^{13}C NMR** (100 MHz, $CDCl_3$): δ 26.7, 28.5, 46.4, 47.2, 48.5, 52.6, 54.2, 55.3, 79.7, 121.2, 125.6, 128.1, 128.9, 130.7, 147.1, 155.9 **LRMS** (ESI+): *m/z* 743.0 ($[M+H]^+$, 35%), 765.2 ($[M+Na]^+$, 100%). **HRMS** (ESI+): *m/z* Calcd. for $C_{40}H_{59}N_{10}O_4^+$ $[M+H]^+$ 743.4715, found 743.4716. **FTIR** (ATR) ν_{max}/cm^{-1}: 2973, 2817, 1684, 1466, 1414, 1365, 1248, 1230, 1161, 766, 696.

*1,8-Bis(2-(4-phenyl-1H-1,2,3-triazol-1-yl)ethyl)-1,4,8,11-tetraazacyclotetradecane **14***

The protected bis-phenyl **17** (243 mg, 0.327 mmol) was dissolved in dioxane (2 mL) and a solution of HCl in dioxane (4.0 M, 1.00 mL, 4.00 mmol) was added. The reaction mixture was stirred at rt for 16 h. The volatiles were removed *in vacuo* and the residue was triturated with EtOAc (2 × 3 mL). The HCl salt was

neutralised with excess Ambersep® 900 hydroxide form resin in MeOH (3 mL) and purified by HPLC (0% to 100% MeCN in H_2O containing 0.1% TFA over 30 min) to afford the TFA salt of **14** as a white solid (173 mg, 69%). **m.p**.: 213 °C (decomp.). ^{1}**H NMR** (400 MHz, D_2O): δ 1.65 – 1.80 (m, 4H), 2.35 – 2.49 (m, 8H), 2.49 – 2.60 (m, 4H), 2.90 – 3.05 (m, 8H), 4.57 (t, 4H, *J* 5.7), 7.36 – 7.44 (m, 2H), 7.44 – 7.52 (m, 4H), 7.76 – 7.82 (m, 4H), 8.39 (s, 2H). ^{13}**C NMR** (100 MHz, D_2O): δ 23.1, 42.8, 43.0, 46.0, 47.0, 49.0, 49.7, 123.2, 125.2, 128.7, 129.2, 129.4, 147.6. **LRMS** (ESI+): *m/z* 543.1 (free base $[M+H]^+$, 100%). **HRMS** (ESI+) *m/z* Calcd. for $C_{30}H_{43}N_{10}^+$ free base $[M+H]^+$ 543.3667, found 543.3665. **FTIR** (ATR) ν_{max}/cm^{-1}: 2856, 1682, 1424, 1199, 1172, 1119, 1073, 829, 770, 718, 695. **Anal.**: Calcd. for $C_{30}H_{42}N_{10}\cdot 3CF_3CO_2H\cdot 3H_2O$: C 48.70, H 5.45, N 15.78; found C 48.50, H 5.35, N 16.06.

*[Zn(**14**)](ClO$_4$)$_2$*

The TFA salt of **14** (94 mg, 0.11 mmol) was stirred in a suspension of Ambersep® 900 hydroxide form resin in EtOH (4 mL) for 10 min. The resin was filtered and $Zn(ClO_4)_2\cdot 6H_2O$ (40 mg, 0.11 mg) was added. The reaction mixture was stirred at reflux for 16 h. The product was isolated by centrifugation, washed with EtOH (2 × 5 mL) and dried *in vacuo*. The product was then redissolved in MeCN (5 mL) and the solution filtered through a 0.2 µm PTFE syringe filter. The solvent was removed and the product was lyophilised to afford the zinc complex of **14** as a white solid (58 mg, 68%). **m.p.**: 214 ºC (decomp.). **LRMS** (ESI+): *m/z* 705.2 ([M+H]+, 100%). **HRMS** (ESI+): Calcd. for $C_{30}H_{42}ClN_{10}O_4Zn^+$ $[M-ClO_4]^+$ 705.2365, found 705.2354. **FTIR** (ATR) ν_{max}/cm^{-1}: 3260, 2875, 1453, 1358, 1331, 1067, 976, 930, 838, 768, 695, 621. **Anal.:** Calcd. for $C_{30}H_{42}Cl_2N_{10}O_8Zn$ C 44.65, H 5.25, N 17.36; found C 44.57, H 5.19, N 17.27.

4. Additional absorption and fluorescence data for 3 and 4

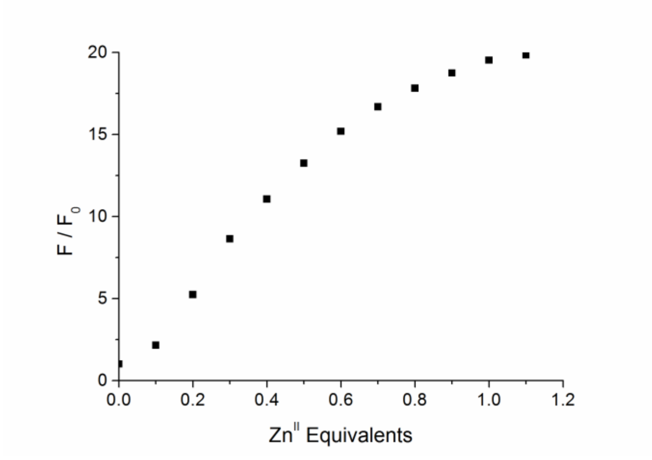

Figure S1: Plot of integrals of emission spectra of **4** in response to increasing Zn^{II} concentration.

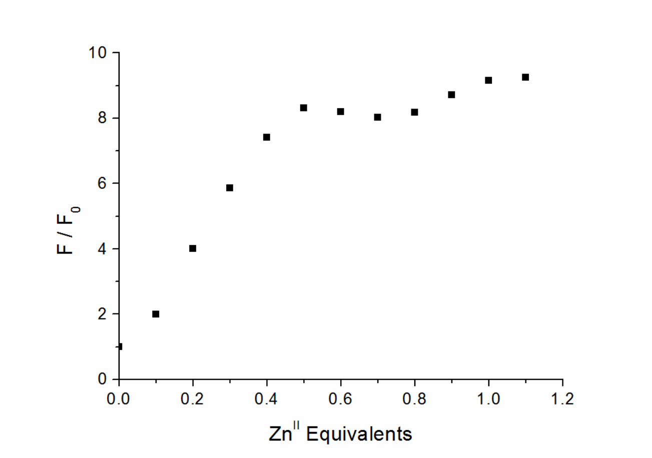

Figure S2: Plot of integrals of emission spectra of **3** in response to increasing Zn^{II} concentration.

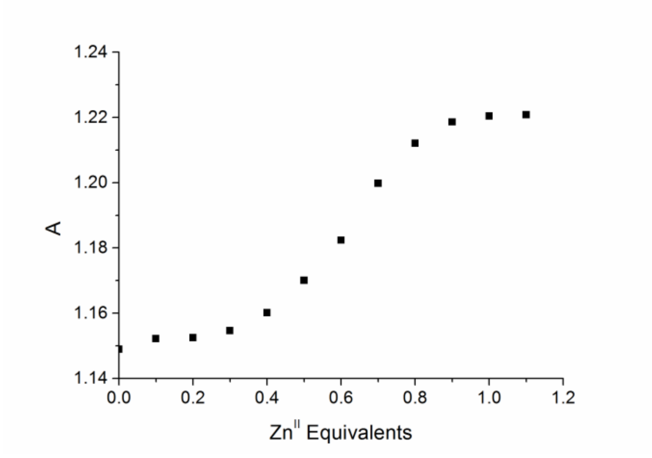

Figure S3: Plot of absorbance at 343 nm of **3** in response to increasing Zn^{II} concentration.

5. Crystal structure determination for [Zn(13)](ClO_4)$_2$ and [Zn(14)](ClO_4)$_2$

Single crystals were attached with Exxon Paratone N to a nylon loop and quenched in a cold nitrogen gas stream from an Oxford Cryosystems Cryostream. A SuperNova Dual equipped with an Atlas detector and employing mirror monochromated Cu (Kα) radiation from a micro-source was used for the X-ray data collections. Data were collected using ω scans at 150(1) Kelvin. Data processing was undertaken with CrysAlisPro[4] and subsequent computations were carried out with WinGX[5] and ShelXle.[6] A multi-scan absorption correction was applied[4] to the data. In both cases the asymmetric unit contains a complex molecule and two perchlorate counterions.

[Zn(**13**)](ClO_4)$_2$: Data were collected from a colourless needle like crystal using ω scans to 151º 2θ, with cell constants obtained from a least squares refinement against 6146 reflections located between 10 and 145º 2θ. The structure was solved in the space group $P2_1$/n(#14) by direct methods with SIR97[7] and extended and refined with SHELXL-2013.[8-9] The non-hydrogen atoms in the asymmetric unit were modelled with anisotropic displacement parameters and a riding atom model with group displacement parameters was used for the hydrogen atoms. The amine hydrogen sites were located in final difference maps. The counterions are linked to the complex molecule by hydrogen bonding to the amine hydrogens. An ORTEP[10-11] depiction of the molecule with 50% displacement ellipsoids is provided in Figure S4.

[Zn(**14**)](ClO_4)$_2$: Data were collected from a colourless blade like crystal using ω scans to 152º 2θ, with cell constants obtained from a least squares refinement against 19032 reflections located between 8 and 152º 2θ. The structure was solved in the space group $P\bar{1}$(#2) by direct methods with SHELXT[12] and extended and refined with SHELXL-2014/7.[8-9] The non-hydrogen atoms in the asymmetric unit were modelled with anisotropic displacement parameters. In general a riding atom model with anisotropic displacement parameters was used for the hydrogen atoms. The amine hydrogen sites were located in final difference maps and modelled with isotropic displacement parameter. One of the counterions is linked to the complex molecule by weak hydrogen bonding to the amine hydrogens. An ORTEP[10-11] depiction of the molecule with 50% displacement ellipsoids is provided in Figure S5.

6. Crystallographic parameters for [Zn(13)](ClO_4)$_2$ and [Zn(14)](ClO_4)$_2$

Table S1: X-ray crystallographic data for complexes [Zn(**13**)](ClO_4)$_2$ and [Zn(**14**)](ClO_4)$_2$.

	[Zn(**13**)](ClO_4)$_2$	[Zn(**14**)](ClO_4)$_2$
empirical formula	$C_{28}H_{38}Cl_2N_{10}O_8Zn$	$C_{30}H_{42}Cl_2N_{10}O_8Zn$
molecular weight	778.95	807.00
crystal system	monoclinic	triclinic
space group	$P2_1$/n	P-1
a (Å)	16.3942(5)	11.4403(4)
b (Å)	8.6714(2)	11.8608(3)
c (Å)	24.0398(10)	15.1765(4)
α (°)	90	73.715(2)
β (°)	104.920(4)	82.790(2)
γ (°)	90	61.259(3)
V (Å^3)	3302.3(2)	1733.06(10)
ρ (calc) (g cm^{-3})	1.567	1.546
Z	4	2
crystal size (mm)	0.190 × 0.026 × 0.017	0.147 × 0.097 × 0.039
crystal colour	colourless	colourless
crystal habit	needle like	blade
Temperature (K)	150(1)	150(1)
λ(Cu Kα) (Å)	1.5418	1.5418
μ(Cu Kα) (mm^{-1})	3.086	2.961
$T_{min,max}$	0.423, 1.00	0.886, 1.00
$2\theta_{max}$ (°)	150.88	152.00
hkl range	-20 20, -10 10, -29 30	-14 13, -14 14, -19 19
N	29296	36415
N_{ind}	6599 (R_{merge} 0.0876)	7062 (R_{merge} 0.0323)
N_{obs} (I > 2σ (I))	4777	6255
N_{var}	442	468
Residuals* $R1(F)$, $wR2(F^2)$	0.0578, 0.1476	0.0460, 0.1258
GoF (all)	1.169	1.379
Residual Extrema (e.Å^{-3})	-0.449, 0.735	-0.643, 1.376

*$R1 = \Sigma||F_O| - |F_C||/\Sigma|F_O|$ for $F_O > 2\sigma(F_O)$; $wR2 = (\Sigma w(F_O^2 - F_C^2)^2/\Sigma(w\ F_C^2)^2)^{1/2}$ all reflections $w = 1/[\sigma^2(F_O^2) + (0.05P)^2 + 1.0P]$ where $P=(F_O^2 + 2F_C^2)/3$

Table S2: Selected atomic distances (Å) and angles (°) for complexes [Zn(**13**)](ClO_4)$_2$ and [Zn(**14**)](ClO_4)$_2$.

	[Zn(**13**)](ClO_4)$_2$	[Zn(**14**)](ClO_4)$_2$
Zn(1)—N(1)	2.049(3)	2.136(2)
Zn(1)—N(2)	2.287(3)	2.241(2)
Zn(1)—N(3)	2.056(3)	2.132(2)
Zn(1)—N(4)	2.196(3)	2.258(2)
Zn(1)—N(5)	2.043(3)	
Zn(1)—N(6)	-	2.204(2)
Zn(1)—N(9)	-	2.236(2)
N(1)— Zn(1)—N(5)	104.91(13)	
N(2)— Zn(1)—N(5)	78.31(12)	
N(3)— Zn(1)—N(5)	121.04(13)	
N(4)— Zn(1)—N(5)	107.33(11)	
N(1)— Zn(1)—N(6)	-	89.91(8)
N(2)— Zn(1)—N(6)	-	87.92(8)
N(3)— Zn(1)—N(6)	-	174.57(9)
N(4)— Zn(1)—N(6)	-	99.33(8)
N(1)— Zn(1)—N(9)		173.88(8)
N(2)— Zn(1)—N(9)	-	99.33(8)
N(3)— Zn(1)—N(9)	-	90.13(10)
N(4)— Zn(1)—N(9)	-	87.01(8)
N(6)—Zn(1)—N(9)	-	84.64(8)

CCDC 1443384 ([Zn(**13**)](ClO_4)$_2$) and 14338555 ([Zn(**14**)](ClO_4)$_2$) contain the supplementary crystallographic data for this paper. These data can be obtained free of charge from The Cambridge Crystallographic Data Centre via www.ccdc.cam.ac.uk/data_request/cif

7. ORTEP depictions of $[Zn(13)](ClO_4)_2$ and $[Zn(14)](ClO_4)$

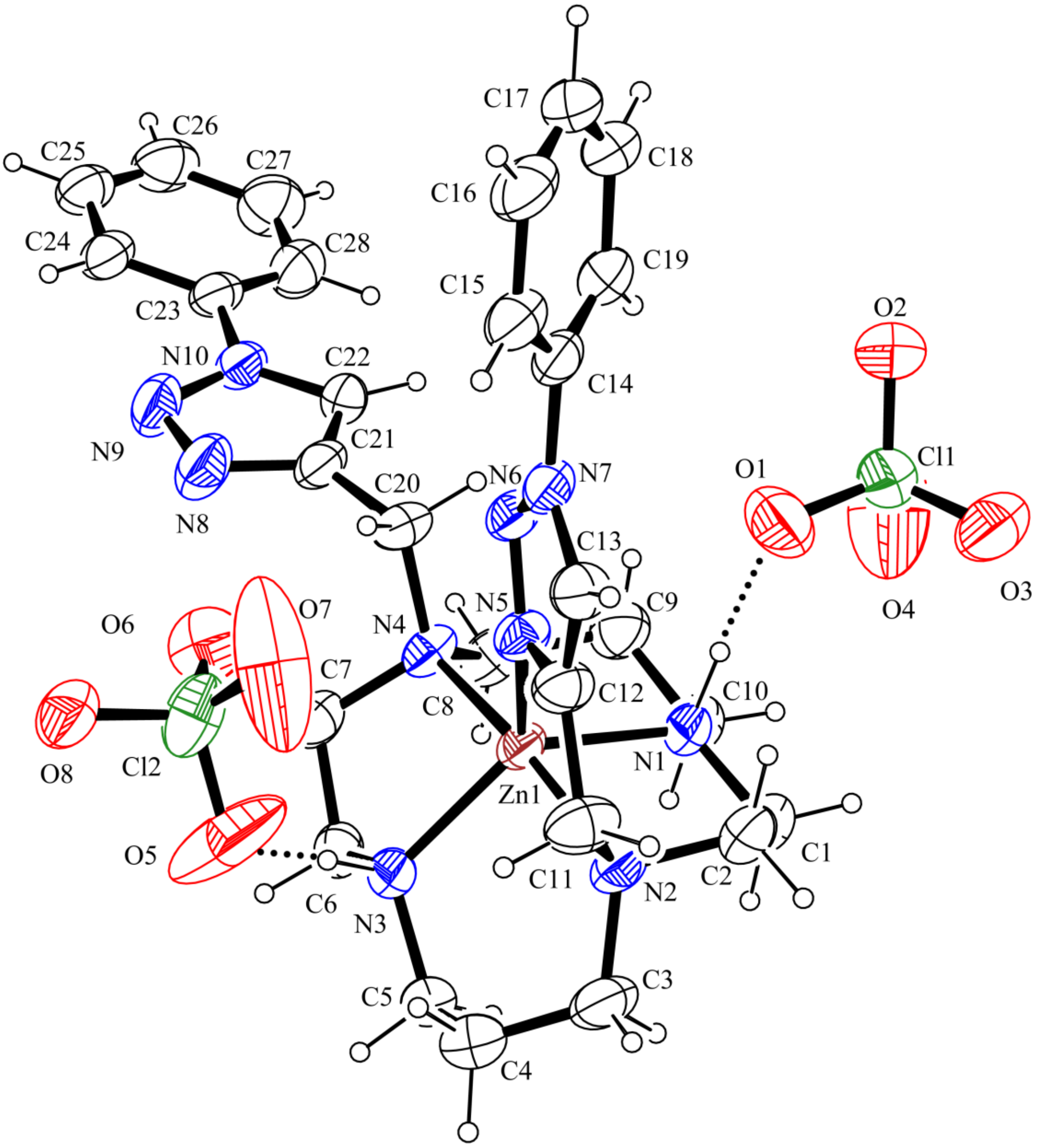

Figure S4: ORTEP depiction of [Zn(**13**)]$(ClO_4)_2$

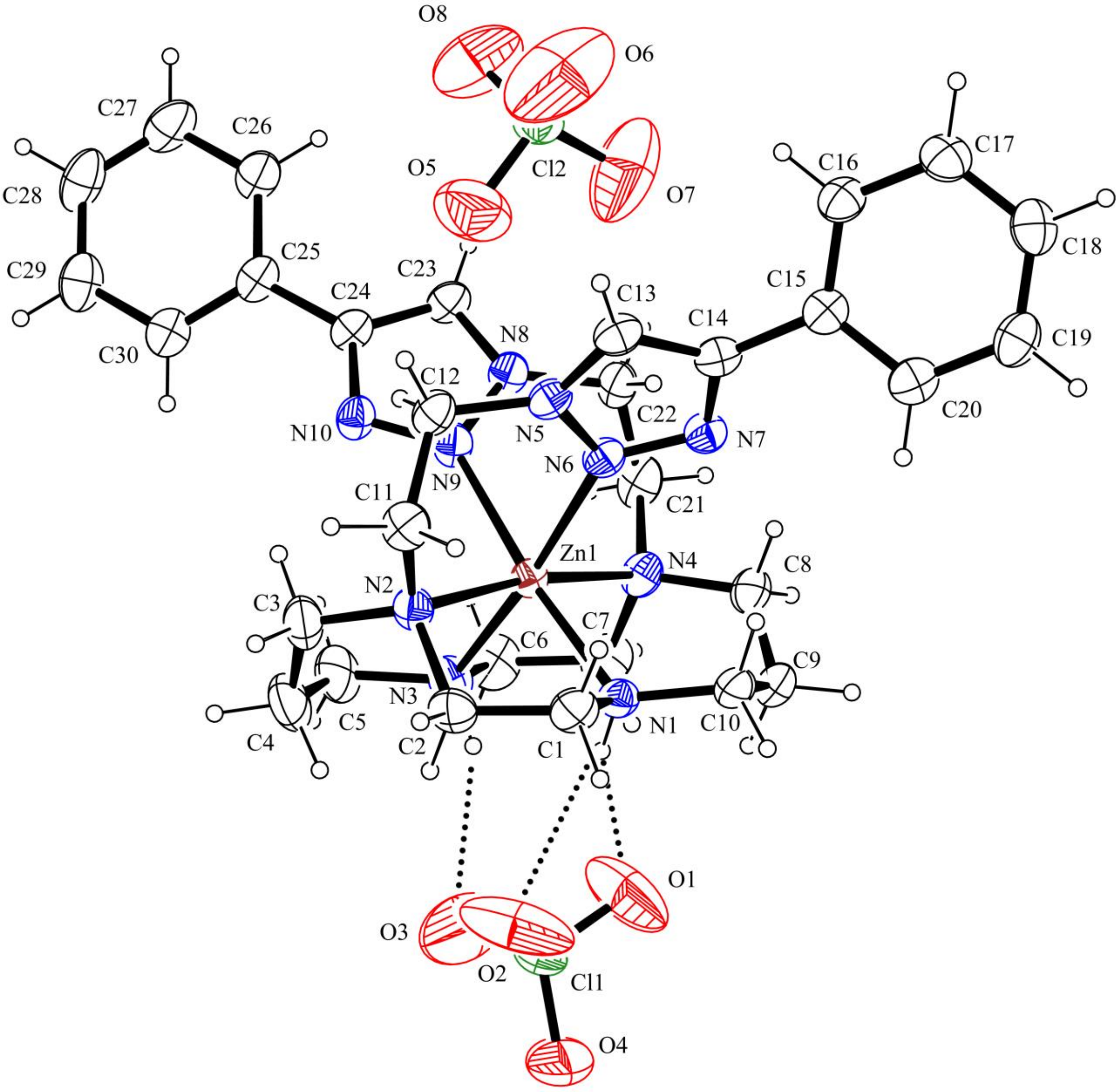

Figure S5: ORTEP depiction of [Zn(**14**)](ClO_4)$_2$.

8. Further Discussion of Crystal Structures

Currently the Cambridge Structural Database (CSD) [13] has nine zinc cyclam complexes that are five coordinate with all of the coordinated atoms being nitrogen. Of these, three are essentially trigonal bipyramidal. The average axial distances of these three are 2.16(3) and 2.200(2) Å; the average equatorial distances are 2.09(3), 2.09(2) and 2.01(4) Å (the last of these being for the non-cyclam ligand, which would otherwise be the apical bond of a square pyramidal complex). The shortest non-cyclam equatorial distance of the three complexes is 1.95(2) Å (CSD refcode SEGHIR); in this case the non-cyclam ligand is isothiocyanate which is not tethered to the macrocycle. The intermediate non-cyclam equatorial distance of the three complexes in the CSD is 2.021(3) Å (DEHPOX) and in this case the coordinating nitrogen is part of a pendant propanamide residue, such that there are three atoms between the coordinating nitrogen and the cyclam. The longest of the non-cyclam equatorial distance of the three complexes is 2.050(3) Å (DEHPEN) and here the coordinating nitrogen is part of a pendant methylpyridyl residue and is separated by two atoms from the cyclam.

The angle subtended by the least squares planes of the triazole and phenyl rings in the [Zn(**14**)]$(ClO_4)_2$ crystal structure is 48.1(1)° for one of the two pendant residues and 28.3(1)° for the second. The angle between the least squares planes of the coordinated triazole and its phenyl substituent in the [Zn(**13**)]$(ClO_4)_2$ crystal structure is 42.1(2)°, while that for the non-coordinated residue is 10.8(2)°. It would appear that pi-pi interactions do not make a significant contribution in determining the nature of the coordination adopted by the complexes. Crystal packing interactions may however play an important role in 'locking in' the coordination mode in the solid state.

The [Zn(**14**)]$(ClO_4)_2$ cyclam nitrogen to zinc bond lengths are 2.241(2), 2.132(2), 2.258(2), 2.136(2) Å and the triazole nitrogen to zinc bond lengths are 2.204(2) and 2.236(2) Å. The CSD currently holds twelve six-coordinate cyclam complexes in which four of the coordinated atoms are cyclam nitrogen atoms and two are non-cyclam nitrogen atoms. In these complexes the average cyclam nitrogen to metal bond lengths are 2.11(2), 2.10(4), 2.12(3) and 2.11(5) Å, while the average non-cyclam nitrogen to metal bond lengths are 2.33(12) and 2.29(9) Å.

Indicative of moderate hydrogen bond interactions, in the [Zn(**13**)]$(ClO_4)_2$ structure the shortest cyclam nitrogen to counterion oxygen distance for one of the perchlorate anions is 3.022(5) Å, while the shortest cyclam nitrogen to perchlorate oxygen distance for the second is 3.032(5) Å. The interaction is weaker in [Zn(**14**)]$(ClO_4)_2$, with cyclam nitrogen to anion oxygen distances of 3.262(5) and 3.273(5) Å.

9. ^{1}H and ^{13}C NMR spectra of 8

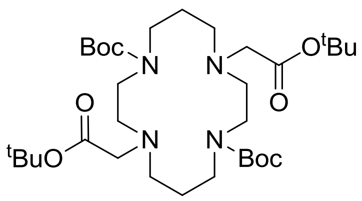

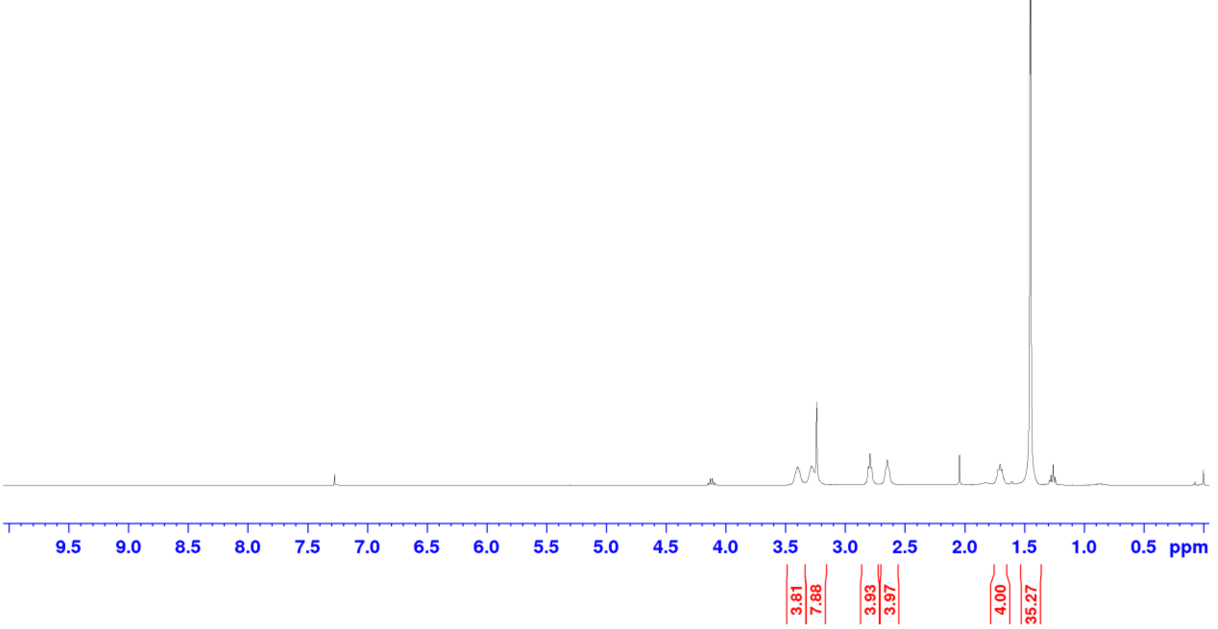

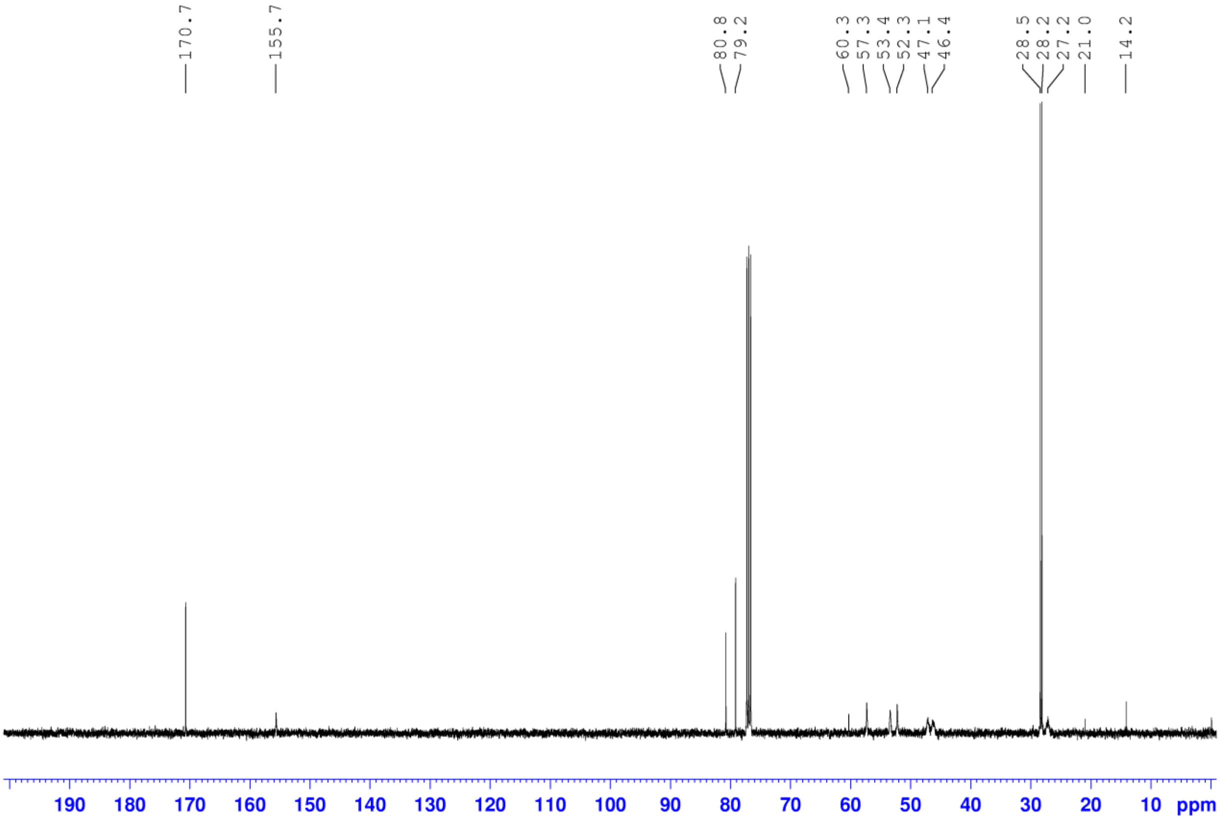

10. ^{1}H and ^{13}C NMR spectra of 9

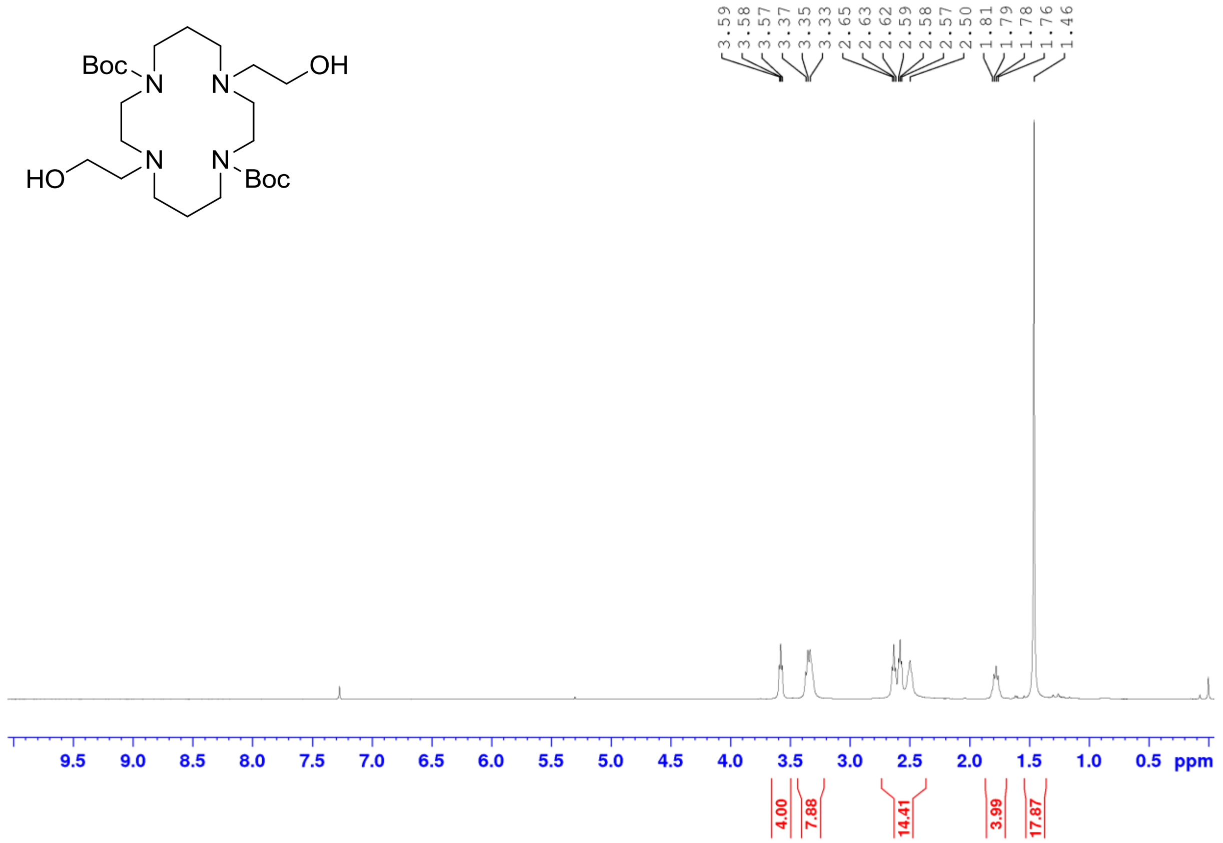

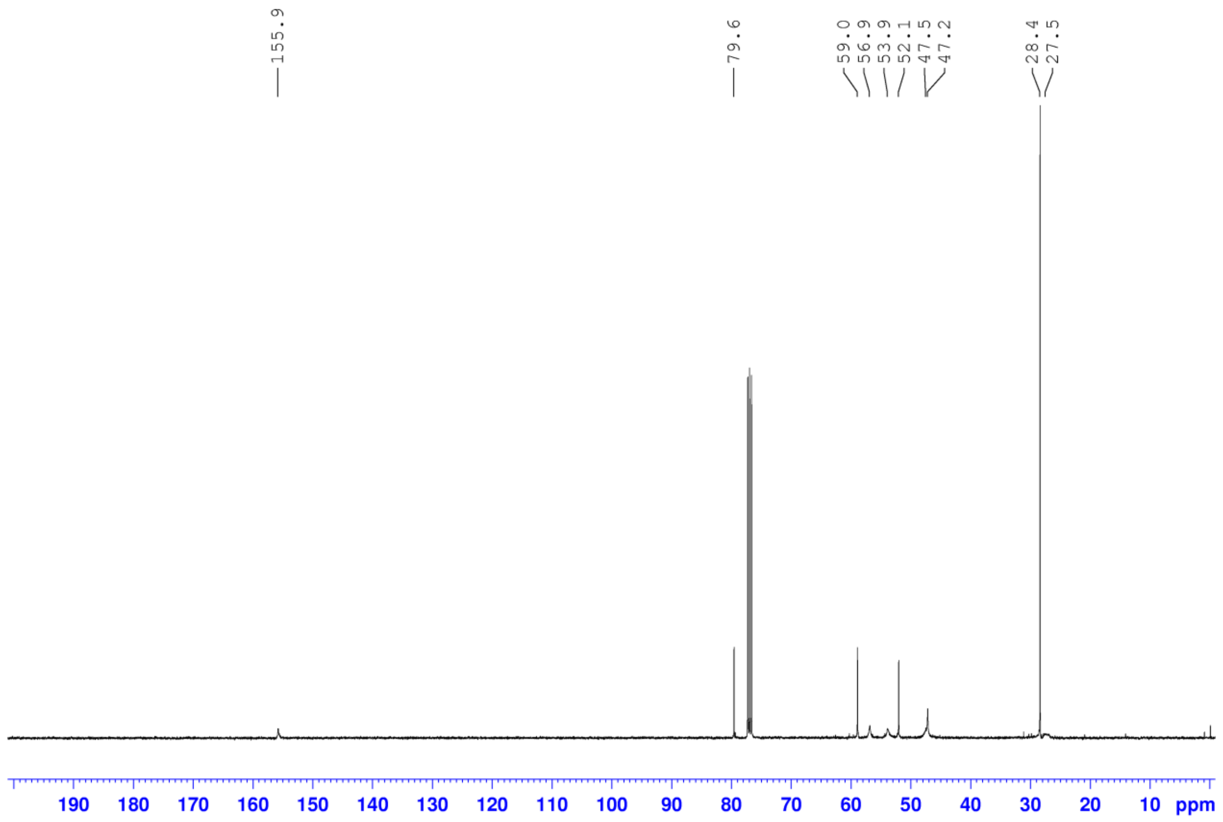

11. ^{1}H and ^{13}C NMR spectra of 10

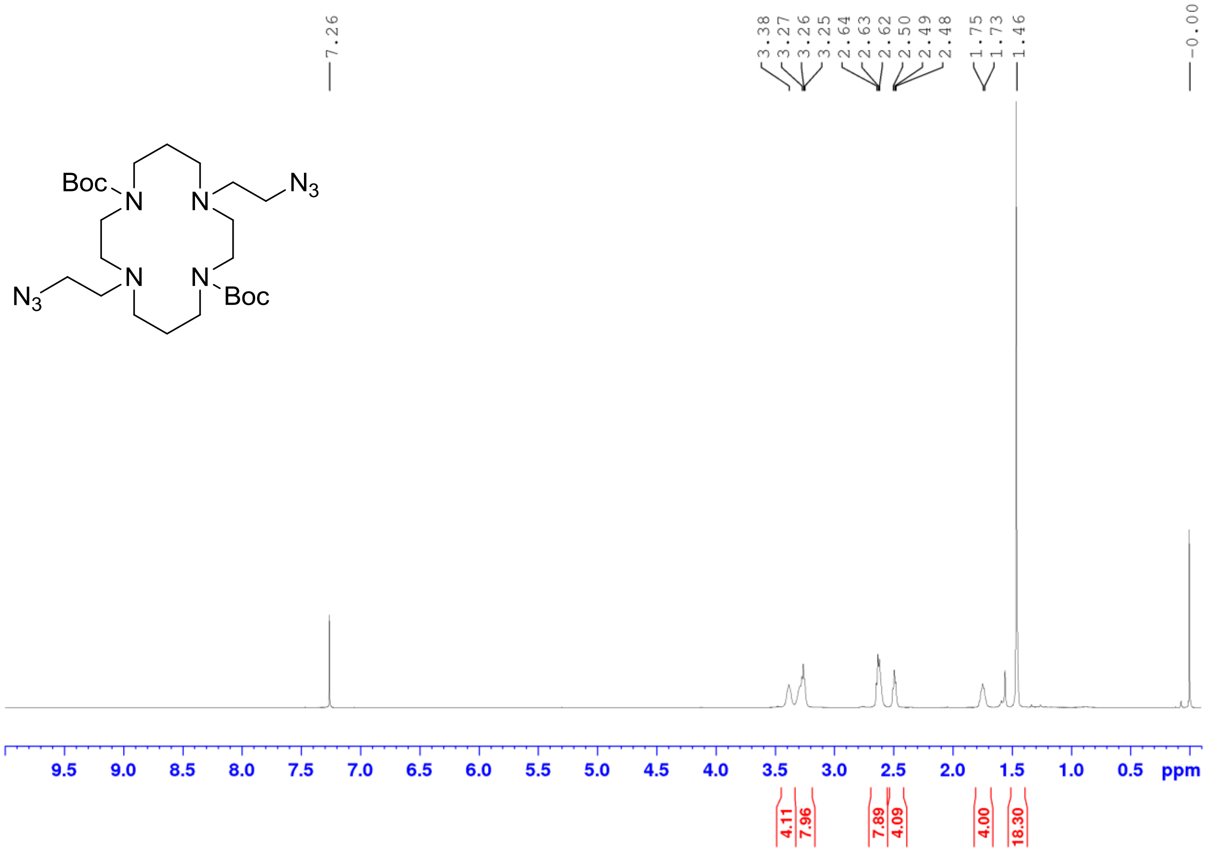

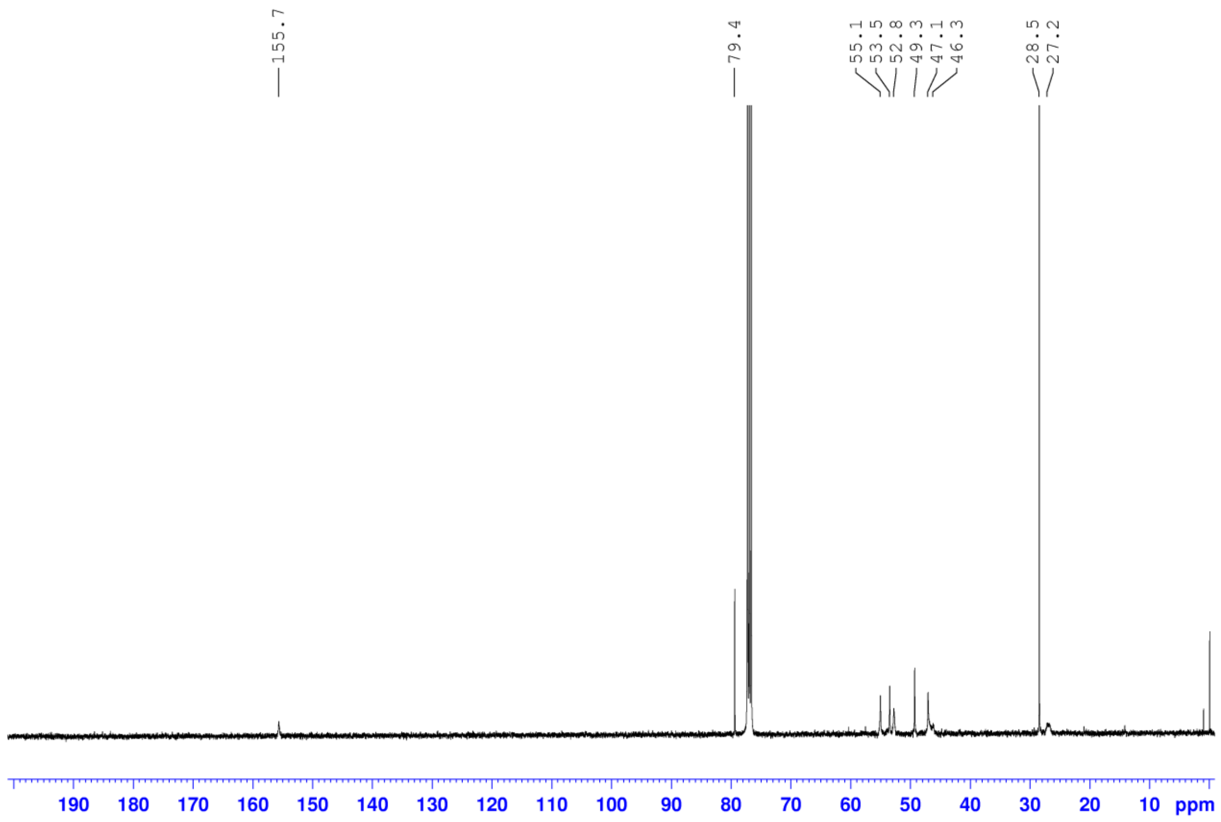

12. ^{1}H and ^{13}C NMR spectra of 12

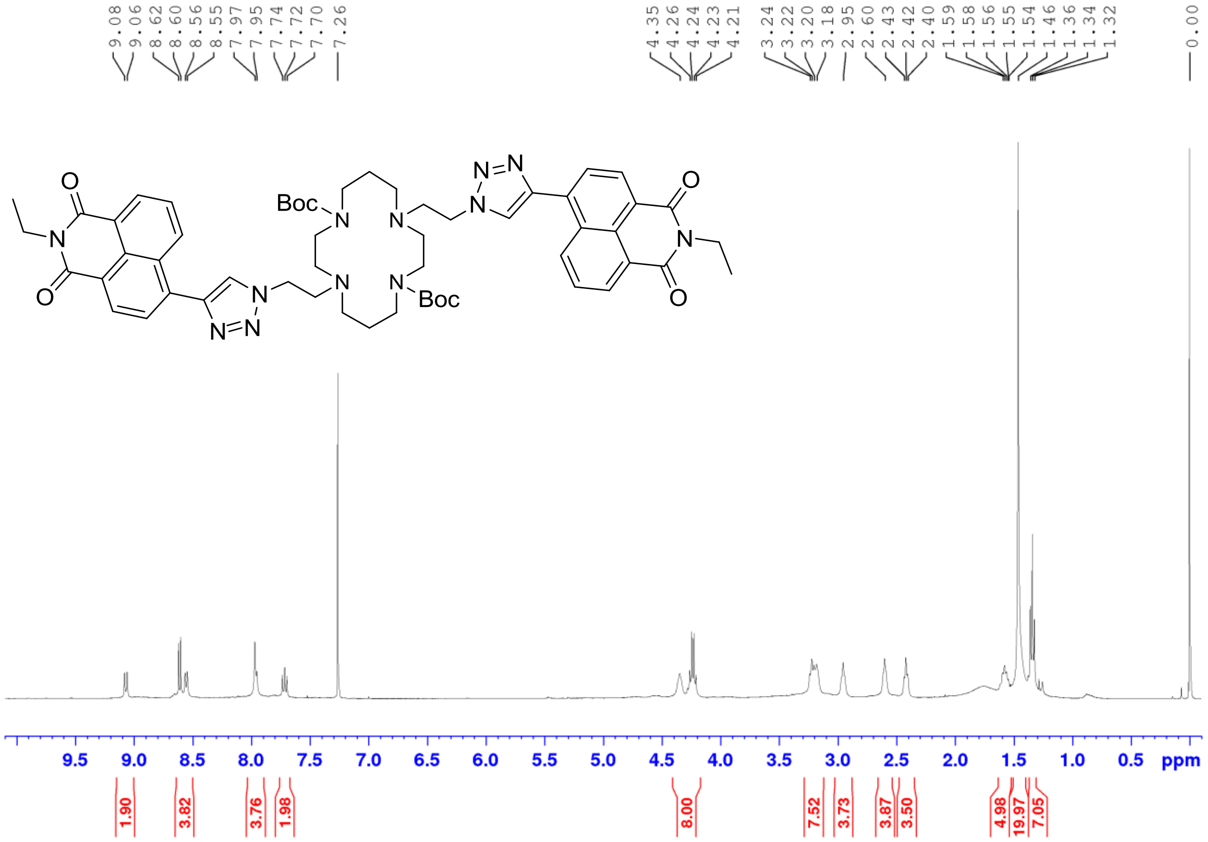

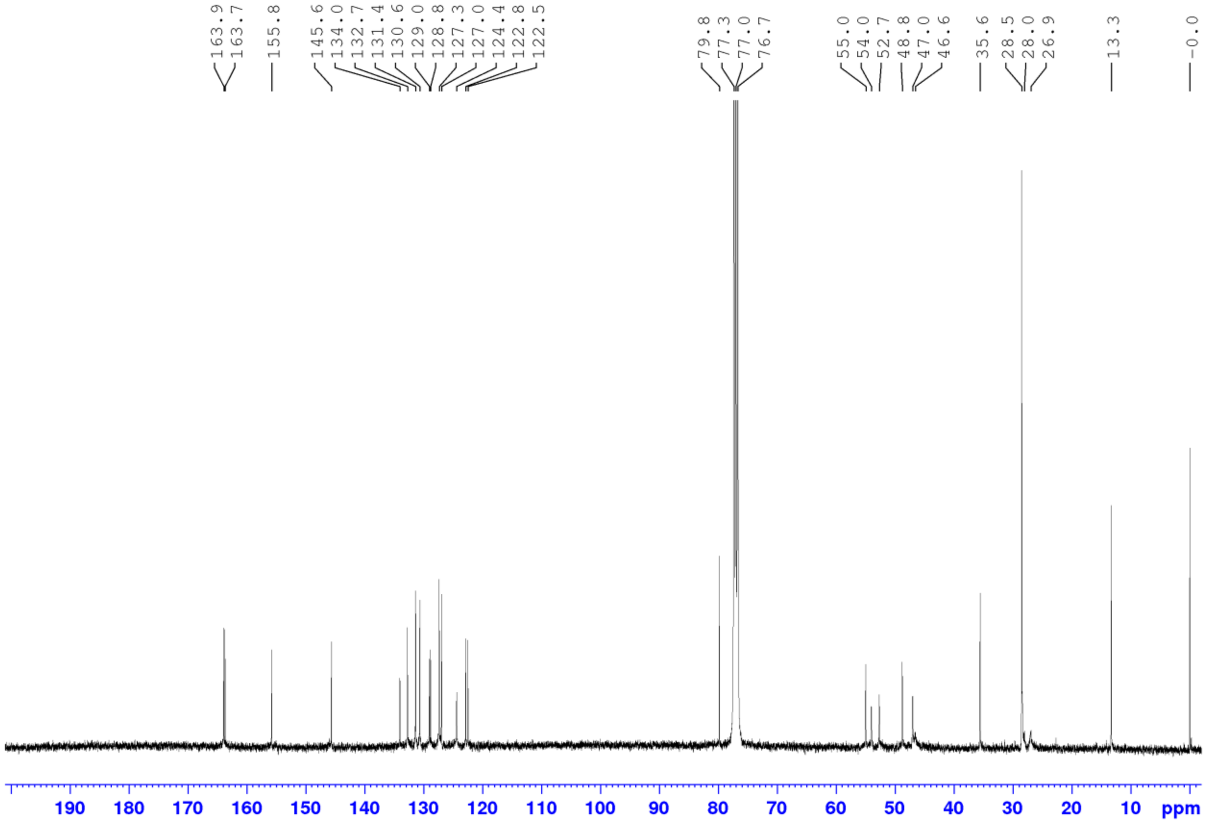

13. ^{1}H and ^{13}C NMR spectra of 4

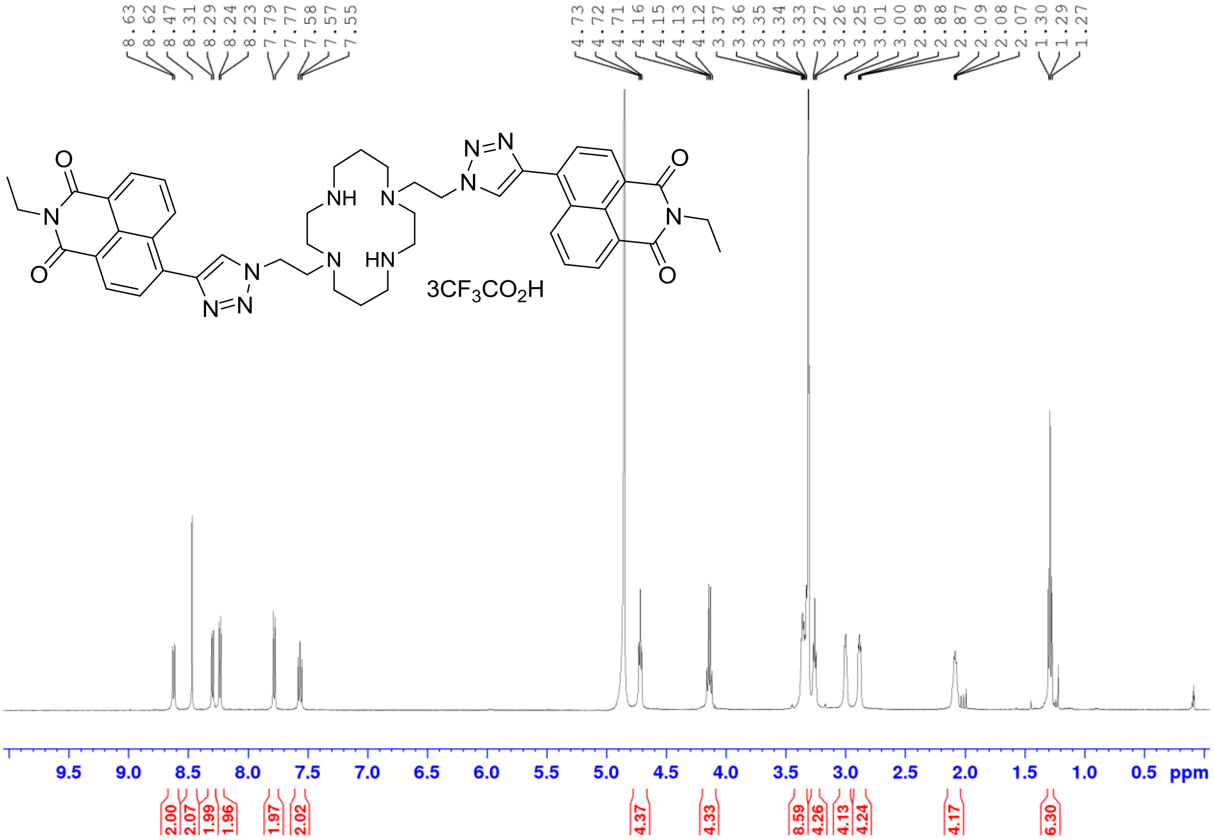

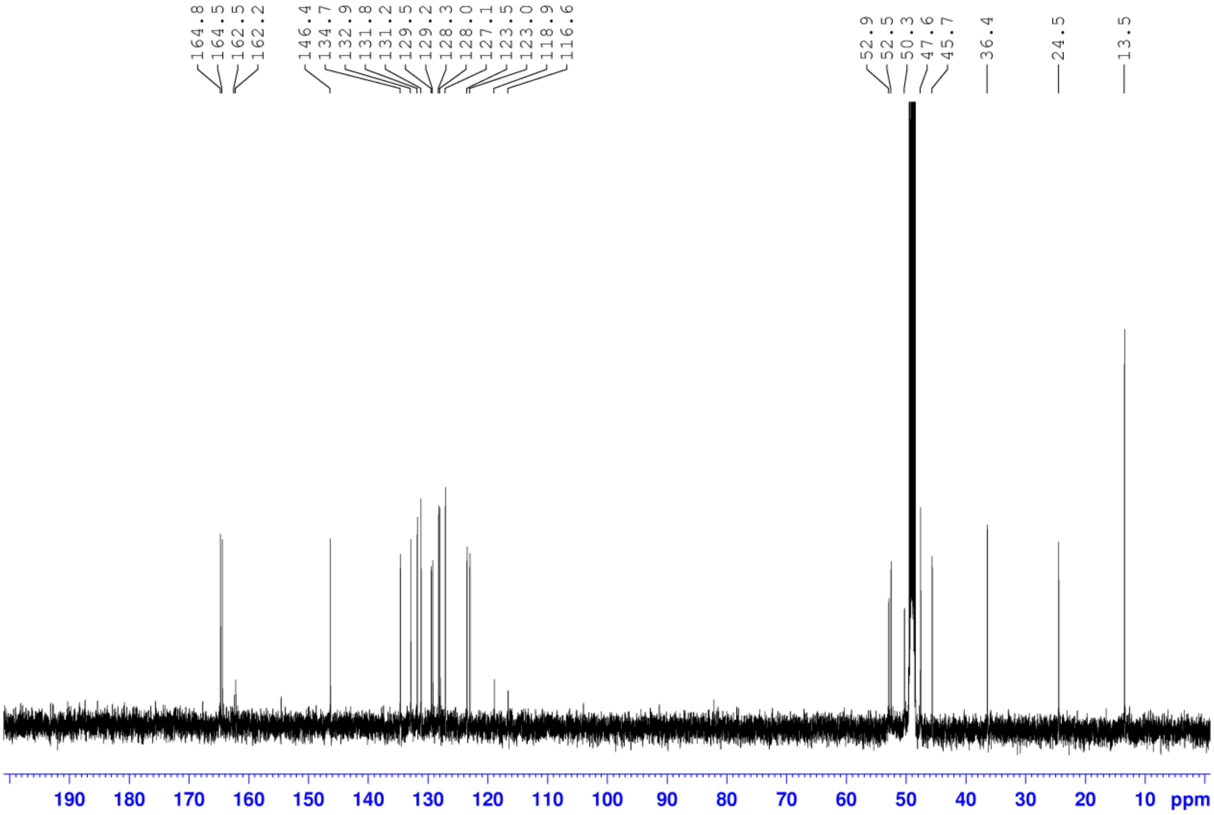

14. Comparison of ^{1}H NMR Spectra of 3 and 3·Zn in 5% DMSO-d_6/CD3CN

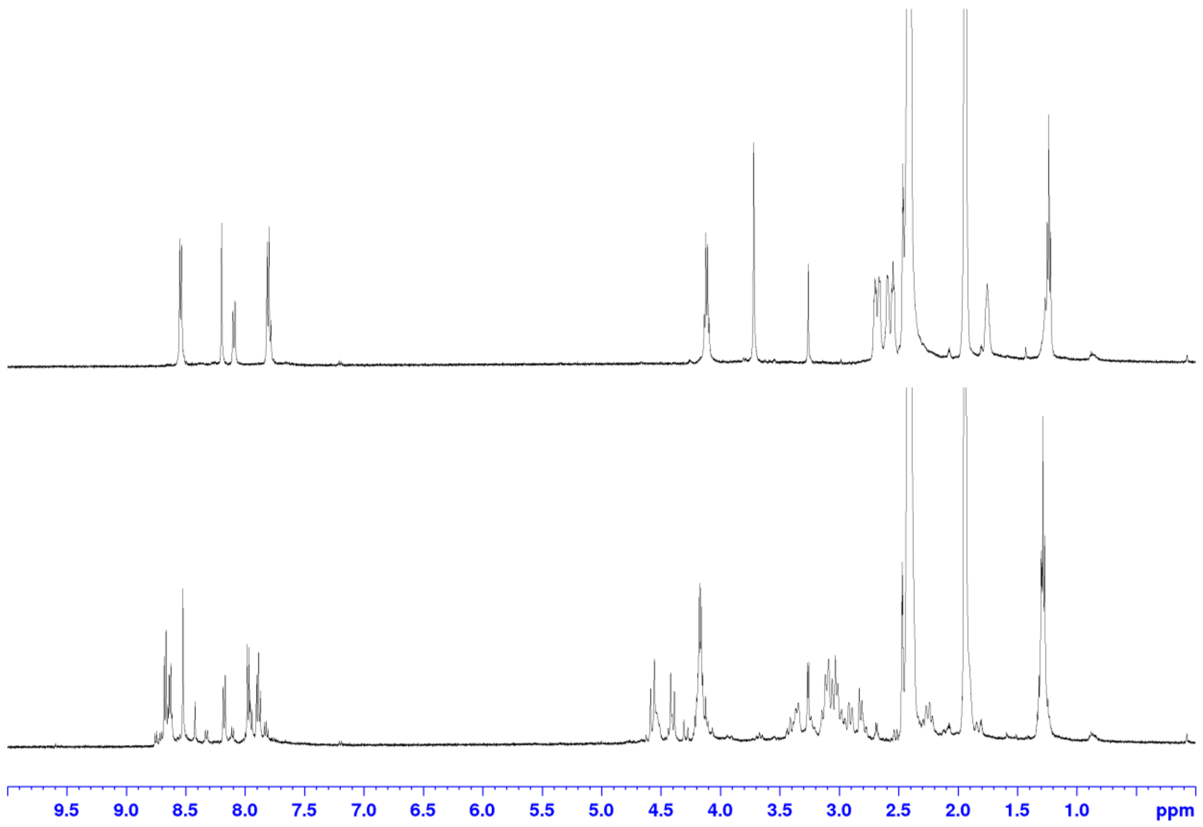

15. Comparison of ^{1}H NMR spectra of 4 and 4·Zn in 5% DMSO-d_6/CD3CN

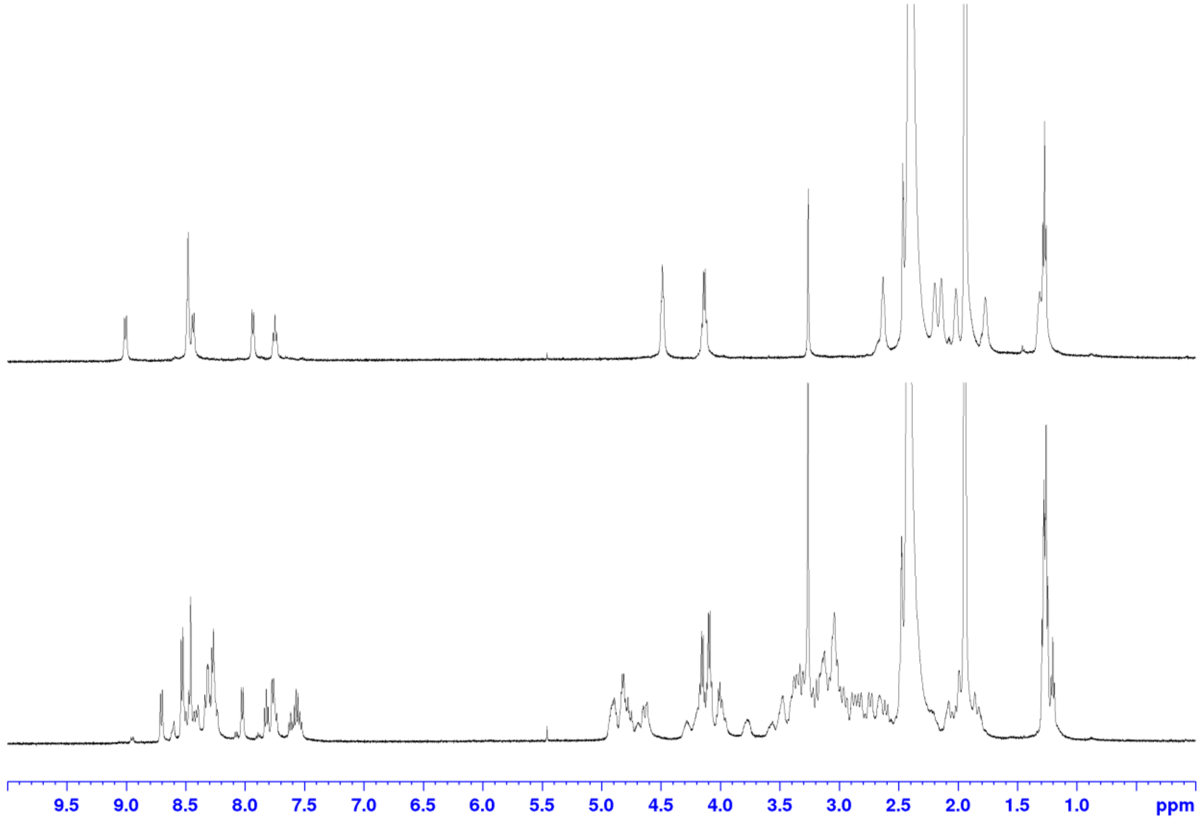

16. References

[1] R. W. Alder, E. Heilbronner, E. Honegger, A. B. McEwen, R. E. Moss, E. Olefirowicz, P. A. Petillo, R. B. Sessions, G. R. Weisman, *J. Am. Chem. Soc.* **1993**, *115*, 6580-6591.

[2] G. Royal, V. Dahaoui-Gindrey, S. Dahaoui, A. Tabard, R. Guilard, P. Pullumbi, C. Lecomte, *Eur. J. Org. Chem.* **1998**, *1998*, 1971-1975.

[3] D. N. Pandya, J. Y. Kim, J. C. Park, H. Lee, P. B. Phapale, W. Kwak, T. H. Choi, G. J. Cheon, Y.-R. Yoon, J. Yoo, *Chem. Commun.* **2010**, *46*, 3517-3519.

[4] CrysAlisPro Version 1.171.37.35, Agilent Technologies, **2015**.

[5] L. J. Farrugia, *J. Appl. Crystallogr.* **1999**, *32*, 837-838.

[6] C. B. Hubschle, G. M. Sheldrick, B. Dittrich, *J. Appl. Crystallogr.* **2011**, *44*, 1281-1284.

[7] A. Altomare, M. C. Burla, M. Camalli, G. L. Cascarano, C. Giacovazzo, A. Guagliardi, A. G. G. Moliterni, G. Polidori, R. Spagna, *J. Appl. Crystallogr.* **1999**, *32*, 115-119.

[8] G. M. Sheldrick, *Acta Crystallogr., Sect. C: Cryst. Struct. Commun.* **2015**, *71*, 3-8.

[9] G. M. Sheldrick, *Acta Crystallogr., Sect. A: Found. Crystallogr.* **2008**, *64*, 112-122.

[10] L. J. Farrugia, *J. Appl. Crystallogr.* **1997**, *30*, 565.

[11] M. N. Burnett, C. K. Johnson, *Acta Crystallogr., Sect. A: Found. Crystallogr.* **1996**, *52*, C93.

[12] G. M. Sheldrick, *Acta Crystallogr., Sect. A: Found. Crystallogr.* **2015**, *71*, 3-8.

[13] F. Allen, *Acta Crystallogr., Sect. B: Struct. Sci* **2002**, *58*, 380-388.
